# Impact of General Practitioner Education on Acceptance of an Adjuvanted Seasonal Influenza Vaccine among Older Adults in England

**DOI:** 10.3390/bs13020130

**Published:** 2023-02-02

**Authors:** Simon de Lusignan, Mansoor Ashraf, Filipa Ferreira, Manasa Tripathy, Ivelina Yonova, Imran Rafi, George Kassianos, Mark Joy

**Affiliations:** 1 Nuffield Department of Primary Care Health Sciences, University of Oxford, Oxford OX2 6GG, UK; 2 Royal College of General Practitioners, Research and Surveillance Centre, London NW1 2FB, UK; 3 Seqirus Ltd., London SL6 8AA, UK; 4 Department of Clinical and Experimental Medicine, University of Surrey, Guildford GU2 7XH, UK; 5 Institute for Medical and Biomedical Education, St George’s University of London, London SW17 0RE, UK

**Keywords:** influenza, influenza vaccines, adjuvanted influenza vaccine, vaccine hesitancy, continuing medical education

## Abstract

Seasonal vaccination against influenza and in-pandemic COVID-19 vaccination are top public health priorities; vaccines are the primary means of reducing infections and also controlling pressures on health systems. During the 2018–2019 influenza season, we conducted a study of the knowledge, attitudes, and behaviours of 159 general practitioners (GPs) and 189 patients aged ≥65 years in England using a combination of qualitative and quantitative approaches to document beliefs about seasonal influenza and seasonal influenza vaccine. GPs were surveyed before and after a continuing medical education (CME) module on influenza disease and vaccination with an adjuvanted trivalent influenza vaccine (aTIV) designed for patients aged ≥65 years, and patients were surveyed before and after a routine visit with a GP who participated in the CME portion of the study. The CME course was associated with significantly increased GP confidence in their ability to address patients’ questions and concerns about influenza disease and vaccination (*p* < 0.001). Patients reported significantly increased confidence in the effectiveness and safety of aTIV after meeting their GP. Overall, 82.2% of the study population were vaccinated against influenza (including 137 patients vaccinated during the GP visit and 15 patients who had been previously vaccinated), a rate higher than the English national average vaccine uptake of 72.0% that season. These findings support the value of GP-patient interactions to foster vaccine acceptance.

## 1. Introduction

Vaccine hesitancy has been topical in the wake of the coronavirus disease 2019 (COVID-19) pandemic. In England, very high rates of vaccination in the first groups to be vaccinated were not repeated in younger age groups [1]. However, vaccine coverage will remain important as COVID-19 develops into an endemic disease worldwide. Influenza, like severe acute respiratory syndrome coronavirus 2 (SARS-CoV-2, the virus that causes COVID-19), is a highly mutable respiratory virus that causes high rates of severe illness and death, especially in older individuals and high-risk groups [2]. During the high-severity 2014–2015 influenza season in the UK, 26,542 deaths among persons ≥65 years of age were attributed to influenza. During the 2017–2018 season, influenza caused 19,525 deaths in the same age group [3,4]. In England during 2017–2018, 25,790 influenza-related hospital admissions in this age group were associated with a cost of £88.4 million [5]. The 2018–2019 influenza season was not considered severe, but influenza nevertheless caused 2939 deaths in those aged ≥65 years in the UK, and in England, 14,955 patients ≥65 years of age were hospitalized at the cost of £52.8 million [3,5]. Moreover, influenza and COVID-19 are associated with long-term cardiovascular complications, extending the impact of these respiratory infections far beyond the acute infection [6,7,8,9].

Vaccines are the most effective means of mitigating the burden of respiratory infections on individual patients and society at large. In the US, an estimated 43,002 deaths and 466,766 hospitalizations occurred among persons aged ≥65 years during the 2017–2018 season [10]. Influenza vaccination during that season prevented an estimated 65,007 hospitalizations and 6796 deaths among those ≥65 years of age, even though vaccine effectiveness at preventing infections in this age group during that season was only 17% [11,12]. Influenza vaccines have also been shown to improve cardiovascular disease outcomes [8]. Despite these benefits, many patients, including older adults, express reluctance to take vaccines. In studies of vaccine hesitancy, reasons for influenza vaccine refusal include the perceptions that influenza vaccines are not effective, are unsafe or poorly tolerated, or themselves cause influenza; in addition, many patients believe that influenza is not a severe enough disease to warrant the risks of vaccination [13,14,15,16,17]. Similar reasons have been given for COVID-19 vaccine hesitancy [18,19,20].

Older patients are particularly vulnerable to respiratory infections because of immunosenescence, or age-related changes in the immune response, which reduce the effectiveness of standard influenza vaccines in this age group [21]. Enhanced vaccines, including one containing a higher influenza antigen dosage and another containing an adjuvant, were developed specifically for older adults, and have shown improved efficacy and effectiveness compared with standard vaccines in persons ≥65 years of age and no increase in reactogenicity post-vaccine compared to other vaccine types [22,23,24,25,26,27,28,29,30].

Based on these findings, the UK Joint Committee on Vaccination and Immunisation (JCVI), US Advisory Committee on Immunization Practices (ACIP), and other agencies recommend giving patients ≥65 years of age either the adjuvanted or the high-dose influenza vaccine [31,32]. The adjuvanted formulation contains MF59^®^, a squalene-based oil-in-water emulsion designed to boost the immune response [33], whereas the high-dose influenza vaccine contains four times the dose of antigen (60 µg) compared with the standard influenza vaccine (15 µg) [23]. The JCVI first recommended immunization with adjuvanted trivalent influenza vaccine (aTIV) for adults aged ≥65 years for the 2018–2019 influenza season; the high-dose influenza vaccine was not yet available [31,34]. However, given that vaccine hesitancy prevents some older adults from accepting *any* influenza vaccine, much less a newly approved one, we conducted a study during the 2018–2019 season to evaluate the impact of healthcare provider education on patients’ acceptance of aTIV in the UK. We present these results to help inform general practitioners’ discussions with their patients during this time of heightened vaccine hesitancy.

## 2. Materials and Methods

### 2.1. Study Design

This study used a combination of qualitative and quantitative approaches to document beliefs about seasonal influenza and seasonal influenza vaccine, assessment of attitudes toward vaccination against influenza, and perceptions of social reference support for vaccination against influenza in general practitioners (GPs, including physicians and other healthcare providers qualified to treat patients) and patients ≥65 years of age. Data were collected during the 2018–2019 influenza season in the UK.

Participating GPs completed two questionnaires that assessed their knowledge and attitudes toward adult immunization, influenza disease, and vaccines. The first questionnaire was administered before and the second immediately after an accredited online continuing medical education (CME) module covering influenza disease and influenza vaccination (including information pertaining to enhanced influenza vaccines for older adults, including aTIV). Physicians completed the CME module on the online site MDBriefCase, during the 2018–2019 influenza season, which provides CME in Europe, Canada, Australia, and the Middle East. The module itself has been archived and is no longer available.

Participating patients ≥65 years of age were recruited by a subset of participating GPs from 12 general practices that made up the patient-focused research arm. Patients were invited to participate during regularly scheduled visits during the influenza season. Patient participants completed two questionnaires assessing their knowledge and attitudes towards adult immunization, influenza disease, and vaccines—one administered before they saw their GP and the second immediately after the visit. All participating patients provided written, informed consent, and the pre- and post-visit questionnaires were administered by a study research nurse.

All participating GPs received a copy of the study report as did patients who wished to receive it.

### 2.2. Data Collection Instruments

The GP questionnaires were developed de novo for the present study and comprised a series of questions based on the information-motivation-behavioural skills (IMB) model, which can be used to evaluate how knowledge-based information, motivation, and behavioural skills influence health behaviour performance. In addition, the subset of 12 GPs whose patients participated in the study answered questions about interactions with their patients related to influenza disease and vaccination.

The questionnaires given to patients took 5–7 min to complete and captured demographic information and prior vaccination history as well as patients’ knowledge and perceptions about influenza disease and vaccinations. The pre-visit questionnaire included open-ended, qualitative, and unprompted questions to assess spontaneously occurring, “top of the head” perceptions about what is good and not good about adult immunization, the identities of groups or persons who influence patients’ decisions regarding immunization (e.g., family members, friends, medical resources), and patients’ levels of understanding about influenza disease and influenza vaccines (Table 1). The post-visit questionnaire included the same series of questions measuring knowledge and perceptions about influenza and vaccines (to measure changes from baseline) as well as additional qualitative, open-ended questions about patients’ knowledge, perceptions, and intention to be vaccinated with aTIV (Table 1).

### 2.3. Study Participants

Participating GPs were required to be members of the Oxford-Royal College of General Practitioners (RCGP) Research and Surveillance Centre (RSC) practice network—one of Europe’s oldest sentinel systems [35]—who participated in the CME program. Eligible patients were adults ≥65 years of age. All participants were required to be able and willing to complete the two sets of questionnaires (either GP or patient questionnaires, as applicable) as well as provide written, informed consent allowing the use of their data for the study. GPs or patients who had participated in previous behavioural research were ineligible for this study.

Of GPs who completed the before and after questionnaires, 12 were invited to recruit patients from their practice, with an expected enrolment of 25–30 patients per study site. This group of GPs first participated in an investigator training webinar prior to recruiting any patients. Each study site then identified patients with scheduled visits and, during pre-visit reminder calls, invited the patients to participate in the study. A study research nurse confirmed patient interest and obtained written, informed consent from each patient participant.

Patients were not renumerated for their participation, but the general practices that served as research centres for the patient arm were renumerated for their time and recruitment of patients.

### 2.4. Study Objectives

The primary objectives of the study were first to assess GPs’ concerns, acceptance, and intention to vaccinate their patients with aTIV after receiving information on the vaccine through an accredited learning program, and second to assess patient concerns, acceptance, and intention to be vaccinated with aTIV after receiving information on the vaccine from a physician or healthcare provider during the course of a routine visit.

Secondary objectives were to describe GPs’ and patients’ knowledge of influenza and their perceptions and attitudes toward adult vaccination, to report any association between the GPs’ and patients’ concerns and the rates of uptake of vaccine in the 2018–2019 influenza season, and to identify gaps in GPs’ or patients’ knowledge about aTIV and the potential impact of these gaps on vaccine uptake.

### 2.5. Statistical Methods

The sample size of 150–200 patients for this descriptive study was determined based on the need to obtain stable estimates of patient knowledge, attitudes, and intentions and sufficient numbers to calculate a two-predictor variable regression analysis of the relationship between attitudes and social norms with intentions to vaccinate.

The Wilcoxon signed-rank test was used to assess changes between pre- and post-visit questions. The association between patients’ attitudes and social norms with intentions to vaccinate before and after the visit with the GP was assessed with binary logistic regression, and odds ratios (OR) were reported with 95% confidence intervals (CI). For the open-ended patient questions, listings with the provided responses were produced in order to be used in post-hoc analyses in which qualitative data analysis methods, including frequency distributions and cross-tabulations, were used to identify keywords in the open-ended responses. These responses were coded by two independent reviewers into a finite number of categories that were defined during the analysis because the categories were data-driven. In the case of disagreement between the two reviewers, a third reviewer was asked to participate in the relevant questions.

Two-tailed tests were performed for all analyses using statistical testing with a significance level of α = 0.05. Summary statistics consisted of the number and percentage of responses in each category for discrete variables. R statistical software (version 3.5) was used to produce all summaries, listings, statistical analyses, and graphs.

## 3. Results

**Figure 1:**
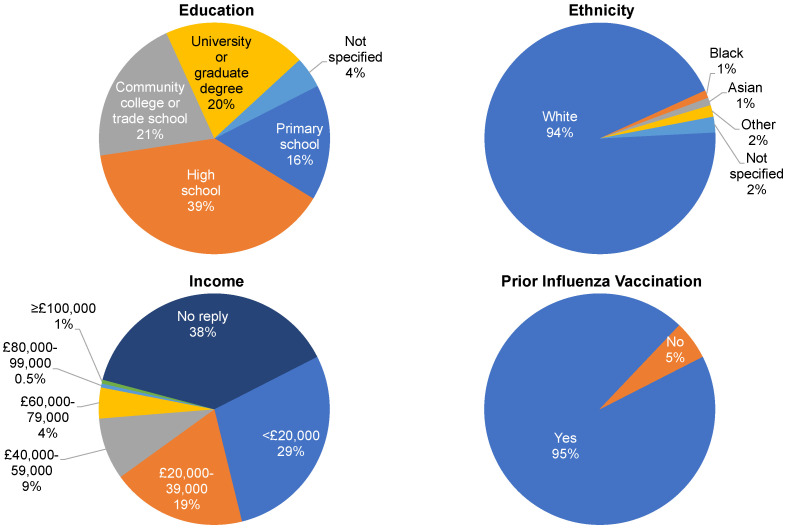
Demographics of the patient population (n = 185). Education refers to the highest level achieved. Income refers to total annual household income. Prior influenza vaccination refers to vaccination during the previous season.

The study participants included 159 GPs and 185 patients who filled out their perspectives before and after questionnaires. The 12 GP participants who recruited and saw the 185 patient participants for routine care also documented their vaccine-related interactions with the patient participants. Figure 1 shows the demographic characteristics of patient participants.

### 3.1. GP Knowledge, Attitudes, and Beliefs about Influenza Disease and Vaccination before and after CME Module

**Figure 2:**
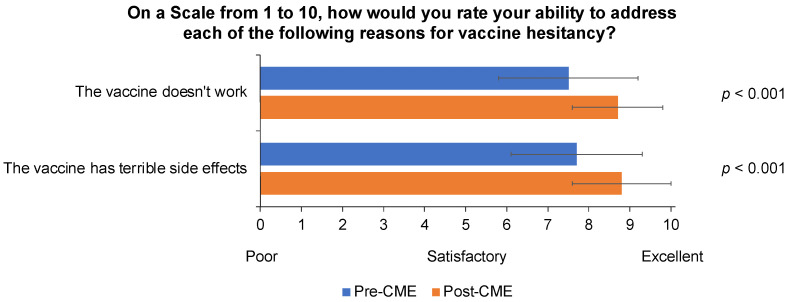
Self-reported rating on a scale of 1–10 of general practitioners’ (n = 159) confidence in their ability to address potential reasons for influenza vaccine hesitancy by patients before and after participating in an accredited continuing medical education (CME) program.

GPs’ self-reported knowledge about influenza disease, aTIV, and JCVI recommendations increased significantly after their participation in the CME module (Figure S1). Both before and after the CME program, most GPs intended to presumptively recommend influenza vaccination to their older patients (93.7% intended to do so before and 96.2% after the CME; *p* = 0.139). A presumptive recommendation was defined as one in which patient acceptance was assumed (e.g., the GP might say, “Time for your flu vaccine. Please roll up your sleeve”). The belief that most patients would accept the GPs’ vaccination recommendations “without much discussion” also did not change significantly (80.5% and 86.8% believed this to be the case before and after CME, respectively; *p* = 0.259), and most GPs anticipated little difficulty in recommending aTIV to their older patients (64.8% before and 67.3% after the CME; *p* = 0.999). Nevertheless, after the CME, more GPs reported a higher level of confidence in being able to address potential reasons for influenza vaccine hesitancy (Figure 2).

**Figure 3:**
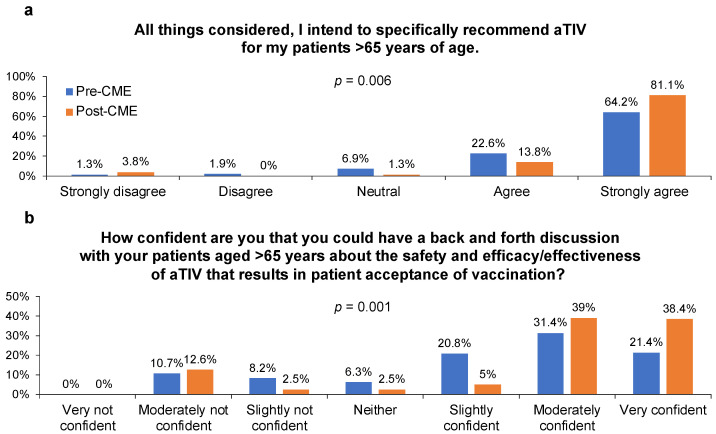
General practitioners’ (n = 159) (**a**) self-reported intention to recommend vaccination with adjuvanted trivalent influenza vaccine (aTIV) for their patients aged ≥65 years before and after participating in an accredited continuing medical education (CME) program and (**b**) confidence in their ability to convince patients to accept vaccination with aTIV.

After the CME, significantly more GPs reported planning to recommend aTIV specifically and most expressed greater confidence in their ability to convince patients to accept vaccination with aTIV during a “back and forth” conversation in which the GP answered patients’ questions about the safety, efficacy, and effectiveness of aTIV (Figure 3). The latter finding may be related to an increased level of confidence in the efficacy and effectiveness of aTIV after the CME, although GPs’ perception of the safety of aTIV did not change significantly (Figure S2).

After the CME program, GPs were asked whether they would presumptively recommend aTIV; 71.7% of responses fell within the range of slightly agree to strongly agree, whereas 20.1% indicated disagreement with a presumptive recommendation for aTIV. Most GPs agreed that presumptive recommendations for aTIV were supported by “important people” (57.2% before and 71.7% after CME; *p* = 0.053), professional membership bodies (61.0% before and 78.0% after CME; *p* = 0.005), and the JCVI (74.8% before and 83.6% after CME; *p* = 0.359). Overall, before and after the CME program, GPs responded more positively than negatively to questions about perceptions of a presumptive recommendation for aTIV for patients aged >65 years for whom aTIV was not contraindicated (Figure S3). Post-CME changes toward even more positive perceptions of “bad vs. good” and “dangerous vs. safe” were statistically significant (Figure S3).

### 3.2. GP Recommendations and Actions Regarding Influenza Vaccination during Patient Visits

**Figure 4:**
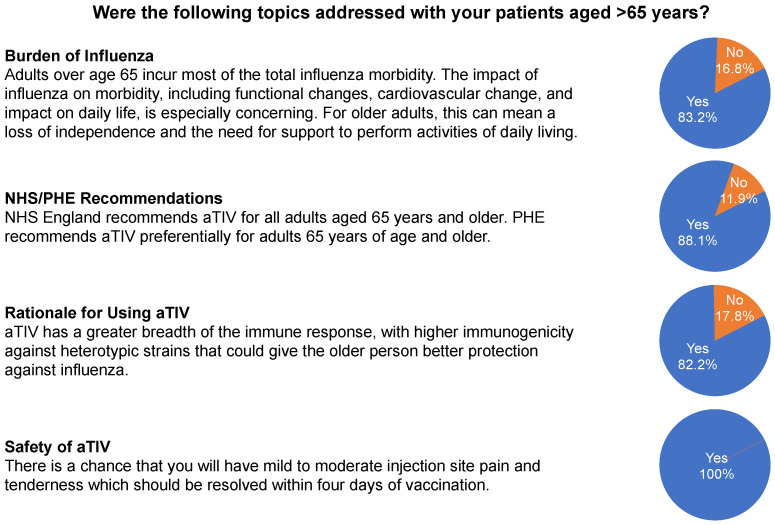
General practitioners’ (n = 12) responses to the patient-interaction questionnaire for 185 patients. NHS, National Health Service; PHE, Public Health England.

GPs who participated in the patient portion of the study (n = 12) reported presumptively recommending aTIV to 116 of 185 patients (62.7%) and not recommending aTIV to 53 patients (28.6%; Table S1). In the majority of patient interactions, GPs reported providing information about the burden of influenza, Public Health England (PHE) recommendations for influenza vaccination, the rationale for using aTIV to help older patients mount a stronger immune response to influenza, and the expected side effects of influenza vaccines (Figure 4).

GPs reported vaccinating 137 of 185 patient participants (74.1%) with aTIV during the routine visit. Of those not vaccinated, 15 patients had already received an influenza vaccination that season and 4 declined consent (Table S1). There was no recorded response for the remaining 29 patients.

### 3.3. Patients’ Knowledge, Attitudes, and Beliefs about Influenza Disease and Vaccination before and after Interactions with Their GP

**Figure 5:**
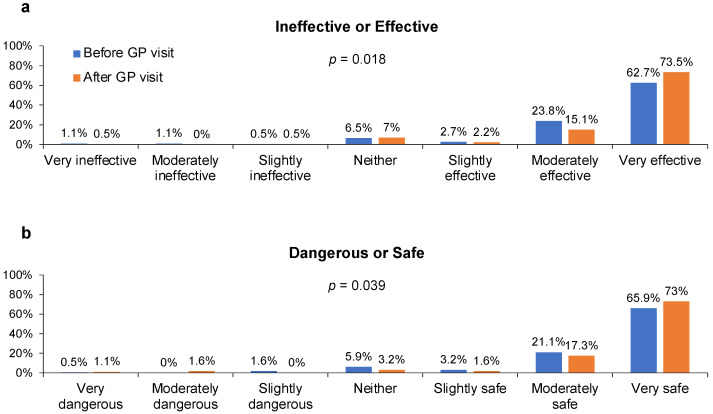
Responses from patients (n = 185) to the statement “Getting a flu vaccine would be (**a**) [ineffective or effective] or (**b**) [dangerous or safe]” (see specific responses along *x*-axes) before and after a routine visit with their GP.

Prior to seeing their GP, 86.4% of 185 patients indicated they planned to be vaccinated against influenza during the 2018–2019 season. Most patients—85.4% before the GP visit and 87.6% afterwards (*p* = 0.101)—also reported that people important to them wanted them to be vaccinated against influenza. A large majority of patients expressed the perception that influenza vaccination was good, wise, effective, safe, and important both before and after the visit with their GP (Figure 5 and Figure S4). However, statistically significant shifts toward more positive perceptions about the effectiveness and safety of influenza vaccines occurred after the GP visit (Figure 5).

In an analysis of the association of attitudes and social norms with vaccination (Table S2), a statistically significant association was seen between patients’ intention to be vaccinated against influenza prior to the GP visit and their perception that vaccination was “bad” or “good” (OR 14.4 [95% CI, 3.7–56.8]; *p* < 0.0001), but patients’ perception that their GP did or did not support influenza vaccination was not significantly associated with their intention to be vaccinated (OR 1.7 [95% CI, 0.9–3.1]; *p* = 0.090). Vaccination with aTIV was modestly associated with patients’ age (OR 1.2 [95% CI, 1.0–1.4]; *p* = 0.039) and the belief that aTIV vaccination would be “wise” (OR 5.2 [95% CI, 2.4–11.2]; *p* < 0.0001).

In their qualitative responses to open-ended questions prior to their GP visit, most patients demonstrated awareness that influenza symptoms could be severe and that influenza might have serious health consequences, particularly for older individuals (Table S3). Many patients expressed the knowledge that influenza vaccines could prevent infections and/or serious illness, and many were also aware that the strains within the vaccine changed annually according to recommendations from health authorities. Stated reasons for an intention to receive the influenza vaccine included a need or desire to strengthen their immune responses to influenza, personal health risks (e.g., asthma, heart disease), and accepted social norms (e.g., “sensible thing to do”) (Table S3). The few patients who planned to refuse the vaccine cited personal preference against vaccination, concerns about vaccine safety, and the belief that they were unlikely to become infected or seriously ill with influenza (Table 2).

After the GP visit, most patients expressed the understanding that aTIV was designed and recommended for older people (some citing specifically those aged ≥65 years), that aTIV boosts the immune system, that it contains an additive (i.e., the adjuvant), and that it offers “better protection” from infection and/or serious disease. Most believed that family members and/or friends would approve of them receiving aTIV; a minority (28/185; 15.1%) anticipated disapproval from others, including family, friends, or associates who were against vaccines generally or believed influenza vaccines gave people the flu, as well as media advocates of anti-vaccine policies. One patient humorously reported that the “undertaker” would be disappointed if the patient received aTIV (Table S4).

Concerns about aTIV safety were expressed by 41/185 (22.2%) of patients, but most of these individuals indicated their belief that adverse effects would be mostly localized injection site reactions of short duration and mild severity. Only one patient expressed concern about long-term side effects from aTIV; the same patient also reported that friends had advised against receiving aTIV. A small group (5/185; 2.7%) were concerned that the influenza viruses contained within aTIV might not match circulating strains and/or that aTIV might fail to prevent influenza infection.

## 4. Discussion

COVID vaccination rates have shown us what might be possible in terms of very high vaccination rates in older adults. We need to continue to rise to this challenge by exploring interventions that might reduce hesitancy and improve uptake.

In this small study of the impact of GP education on patient acceptance of influenza vaccination, GPs gained confidence in their ability to inform patients and address their concerns about influenza and influenza vaccines, even though the educational program did not have a significant impact on the GPs’ knowledge of influenza or intention to vaccinate their patients (which were already high before the CME module). Greater GP confidence was associated with patients’ increased trust in the effectiveness and safety of influenza vaccines. In addition, patients reported few concerns about the adjuvanted influenza vaccine. At the time of this study, aTIV was newly recommended for older adults to provide improved protection from influenza infection, especially with A(H3N2) after low vaccine effectiveness against this strain seen during the 2017–2018 influenza season and the notably increased burden of A(H3N2) in the ≥65-year population [36,37]. At the time, the high-dose influenza vaccine was not yet available in the UK [34]. Altogether, these findings suggest that CME can have a positive impact on GPs’ communication with their patients, which in turn can foster improved adherence to the GP’s recommendations.

The IMB model used in this study is well-established and provides a solid foundation for questionnaire development pertaining to vaccination behaviours and vaccination intentions [38,39]. The reasons underlying vaccine hesitancy in this study were consistent with previous studies. In a meta-analysis of 58 studies of influenza vaccine hesitancy in patients aged ≥65 years, patients’ rationales for vaccine acceptance and refusal mirrored those given by participants in this study, including perceived low susceptibility to influenza or its complications, concerns about influenza vaccine safety or tolerability, previous negative experiences with influenza vaccination, fear of injection pain or other side effects, doubts about vaccine effectiveness, and fear of catching influenza from the vaccine [17]. Multiple studies cited in the meta-analysis, however, demonstrated the positive influence of healthcare providers’ advice and recommendations on vaccine acceptance and also supported the positive (and negative) influence of family members and friends [40,41,42,43,44], as seen in our results.

Vaccine acceptance rates tend to be higher among older than younger adults [13,14,15,18,19,20]. This may be due to cultural factors influencing older individuals’ trust in healthcare providers’ recommendations but also might be attributed to outreach efforts by the National Health Service (NHS) specifically aimed at increasing vaccination against influenza during the COVID-19 pandemic [45,46]. When this study was conducted in 2018–2019, vaccine uptake among general practice patients aged ≥65 years in England was 72.0% and in the following season (2019–2020) was 72.4% [47,48]. After COVID-19 began, vaccination rates in this age group increased to 80.9% and 82.3% in the 2020–2021 and 2021–2022 influenza seasons, respectively [49]. These figures support the effectiveness of public health campaigns, especially when coupled with GP advocacy for vaccines. Our findings also support the value of the GP’s role. In this study, 82.2% of the study population were vaccinated against influenza, including 137 patients vaccinated during the GP visit and 15 patients who had been previously vaccinated.

This study has some limitations. Conclusions from this study are associations and do not imply causation. Practices who joined the primary care surveillance system may have higher levels of interest in vaccination, although generally the vaccination rates in the network were not greatly different from national levels of vaccination. It is possible that there was selection bias, and people who volunteered for this study were actually more favourably disposed toward vaccination—95% of participating patients were white, and vaccination acceptance rates tend to be higher in this population [50]. Finally, the survey instruments used were developed de novo for this study and were not formally validated, which may limit the interpretation of the results.

## 5. Conclusions

In this study of GPs’ and older adult patients’ knowledge, attitudes, and beliefs about influenza disease and vaccines, GPs and patients alike reported high levels of understanding that influenza can have serious consequences and that influenza vaccines are a valuable tool to help prevent infections and lessen disease burden. Participation in an influenza CME module increased GPs’ confidence in their ability to answer patients’ questions and address their concerns about influenza and influenza vaccination. After routine visits with GPs who participated in the educational program, patients reported higher levels of confidence in the effectiveness and safety of an adjuvanted influenza vaccine. These findings support the importance and value of the GP’s role in encouraging vaccine acceptance among patients ≥65 years of age.

## Figures and Tables

**Table 1:** Qualitative, open-ended questions included in questionnaires patients filled out before and after a visit with their GP.

Before GP Visit	After GP Visit
What do you know about seasonal flu infection?	What do you know about seasonal flu infection?
What do you know about seasonal flu vaccination?	What do you know about the adjuvanted seasonal flu vaccine?
Last winter, did you get a seasonal flu vaccine? Why?	What concerns do you have about getting the adjuvanted seasonal flu vaccine?
This current winter, will you get a seasonal flu vaccine? Why?	Some of the good things about getting the adjuvanted seasonal flu vaccine
	Some of the bad things about getting the adjuvanted seasonal flu vaccine
	Who would approve of you getting the adjuvanted seasonal flu vaccine?
	Who would disapprove of you getting the adjuvanted seasonal flu vaccine?

**Table 2:** Individual patients’ negative responses to question, “This current winter, will you get a seasonal flu vaccine?”

Reasons for Refusal
Because I didn’t want it and I don’t normally get flu
Had once and had a bad reaction
Didn’t think it was necessary. Not keen on needles
Because I had whooping cough vaccine
I consider myself for my age really fit and able to cope. Some elderly peoplemay need it; I could fight it off
Don’t think about flu so won’t bother
Using homeopathy; some side effects for some people

## Data Availability

All data obtained in this study are reported in the article and supplementary tables and figures.

## Supplementary Materials

The following supporting information can be downloaded at: https://www.mdpi.com/article/10.3390/bs13020130/s1, Figure S1: General practitioners’ (GPs) responses to knowledge-based questions before and after participating in an accredited continuing medical education (CME) program; Figure S2: General practitioners’ level of confidence in the safety (a) and efficacy/effectiveness (b) of adjuvanted trivalent influenza vaccine (aTIV) for adults >65 years of age before and after participating in an accredited continuing medical education (CME) program; Figure S3: General practitioners’ responses to the statement, “Presumptively recommending adjuvanted seasonal influenza vaccine (aTIV) to all of my older adult patients (>65 years of age) in whom influenza vaccination is not contraindicated would be”: (a) bad or good, (b) foolish or wise, (c) ineffective or effective, (d) dangerous or safe, (e) unimportant or important, (f) a waste of time or not a waste of time; Figure S4: Patients’ responses to the statement, “Getting a flu vaccine would be”: (a) bad or good, (b) foolish or wise, (c) unimportant or important; Table S1: Reasons reported by general practitioners (n = 12) for not presumptively recommending adjuvanted trivalent influenza vaccine (aTIV) or not administering aTIV to patient participants >65 years of age; Table S2: Association of attitudes and social norms with the intention to vaccinate and vaccination with aTIV; Table S3: Responses to open-ended patient questions answered prior to a visit with healthcare provider/physician (each row represents a different patient’s response); Table S4: Responses to open-ended patient questions answered after visit with healthcare provider/physician (each row represents a different patient’s response). The protocol and NIHR ethics approval documents are also available upon request.
