# Supplementary material for: Impact of General Practitioner Education on Acceptance of an Adjuvanted Seasonal Influenza Vaccine among Older Adults in England
Source: Behav Sci (Basel). 2023 Feb 2;13(2):130. doi: 10.3390/bs13020130 (PMC9952828; doi:10.3390/bs13020130)
Supplement: Supplementary file 1 [file behavsci-13-00130-s001.zip › behavsci-2128420-supplementary.pdf]

Figure S1. General practitioners' (GPs) responses to knowledge-based questions before and after participating in an accredited continuing medical education (CME) program.

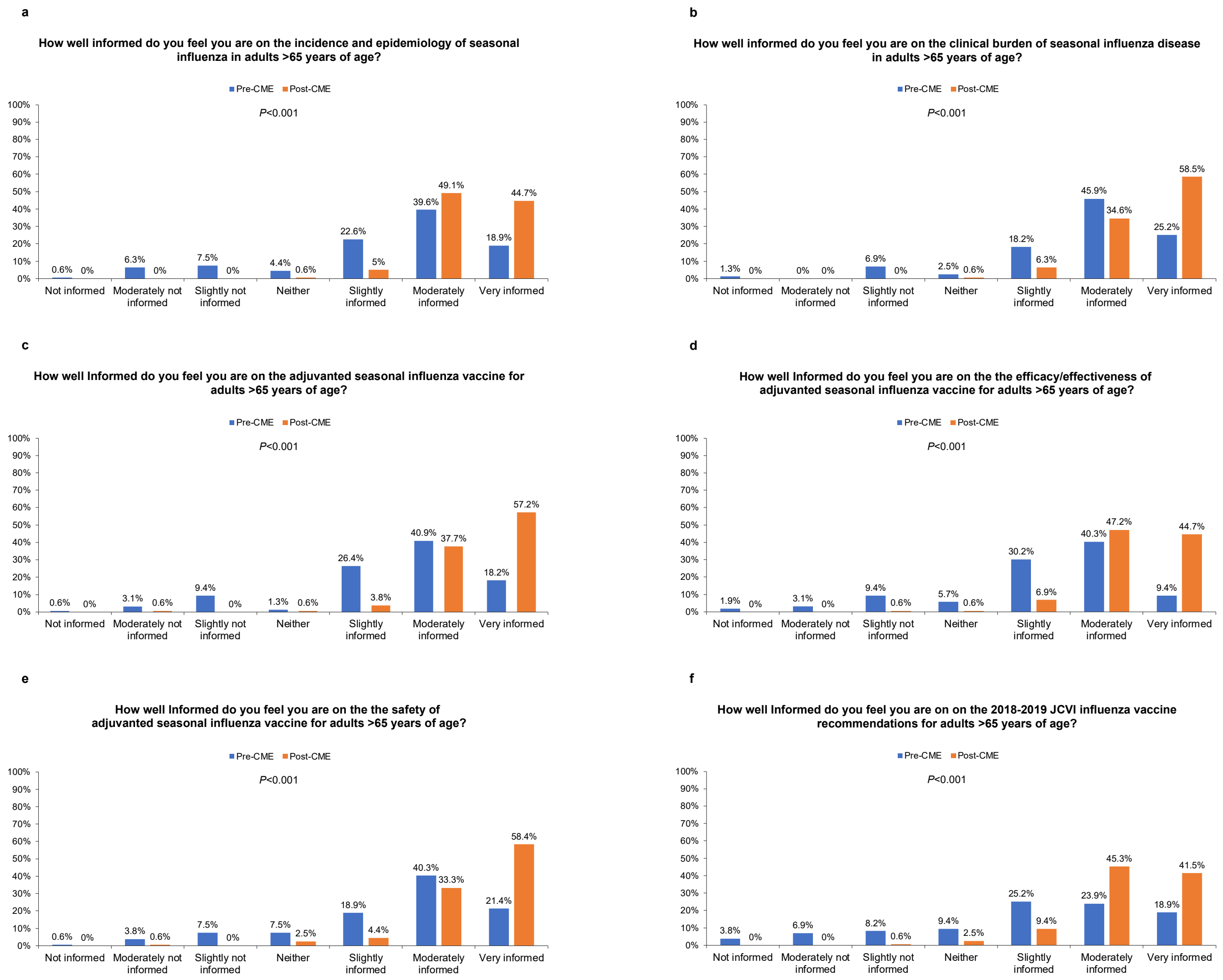

Figure S2. General practitioners' level of confidence in the safety (**a**) and efficacy/effectiveness (**b**) of adjuvanted trivalent influenza vaccine (aTIV) for adults >65 years of age before and after participating in an accredited continuing medical education (CME) program.

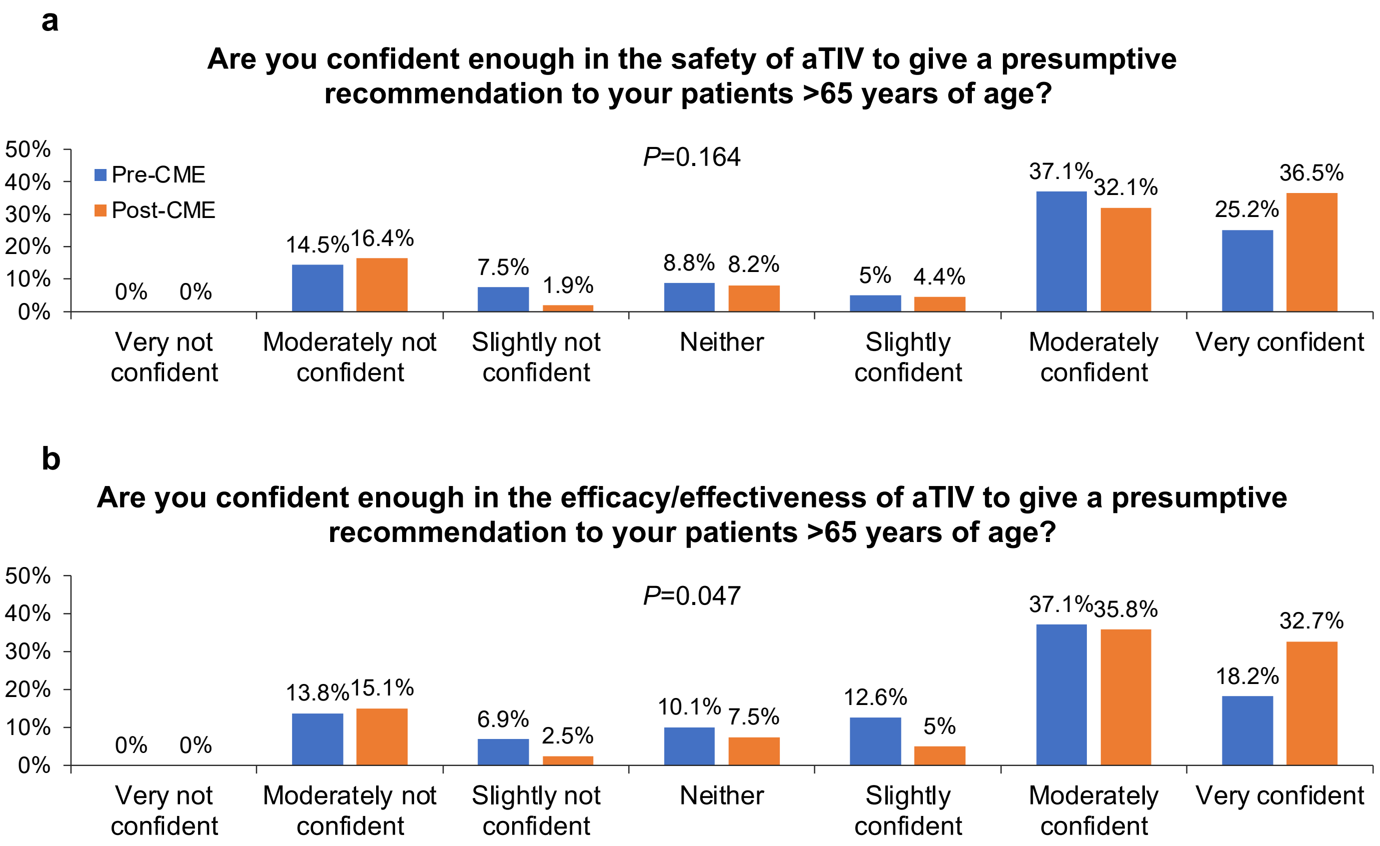

Figure S3. General practitioners' responses to the statement, "Presumptively recommending adjuvanted seasonal influenza vaccine (aTIV) to all of my older adult patients (>65 years of age) in whom influenza vaccination is not contraindicated would be": (**a**) bad or good, (**b**) foolish or wise, (**c**) ineffective or effective, (**d**) dangerous or safe, (**e**) unimportant or important, (**f**) a waste of time or not a waste of time.

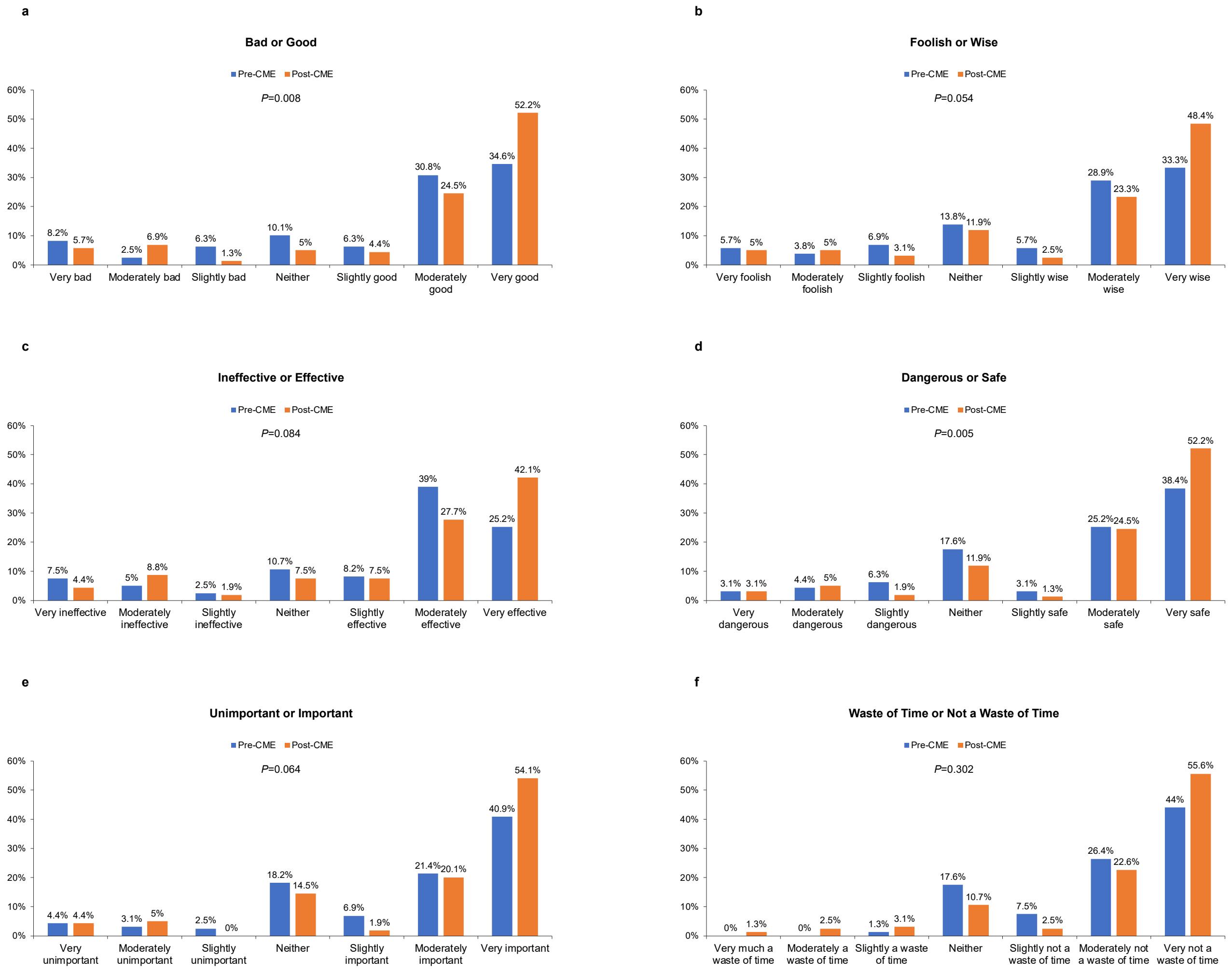

Figure S4. Patients' responses to the statement, "Getting a flu vaccine would be": (**a**) bad or good, (**b**) foolish or wise, (**c**) unimportant or important.

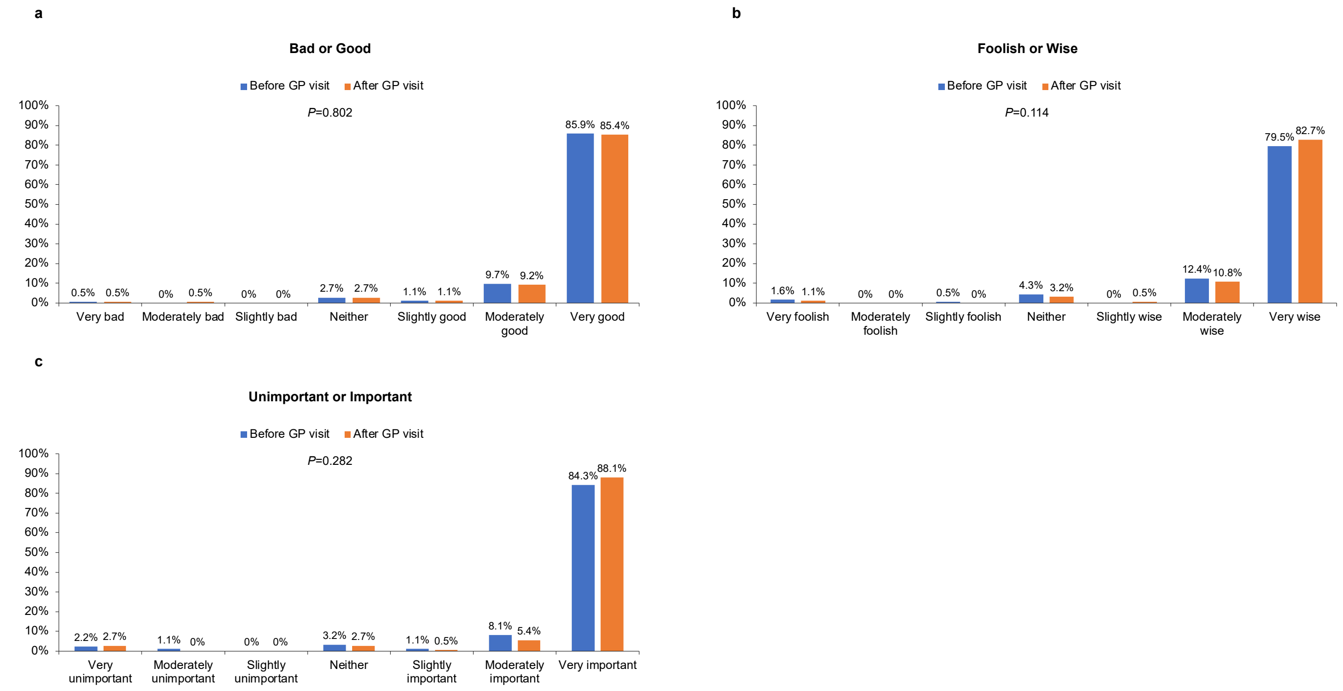

Table S1. Reasons reported by general practitioners (n = 12) for not presumptively recommending adjuvanted trivalent influenza vaccine (aTIV) or not administering aTIV to patient participants >65 years of age.

Reason	Patient participants, n (%)
**No presumptive recommendation**	**n=53**
Already had influenza vaccine	17 (32.1)
Consent	8 (15.1)
Retrospective study (i.e., already vaccinated)	20 (37.7)
Carer or family member refused consent	1 (1.9)
Influenza season over	1 (1.9)
Other	6 (11.3)
**No aTIV vaccination**	**n=22**
Already had influenza vaccine	15 (68.2)
Declined/no consent	4 (18.2)
Other	3 (13.6)

Table S2. Association of attitudes and social norms with the intention to vaccinate and vaccination with aTIV.[1.]

	B	SE	Wald	df	Sig. (*P* value)	Odds ratio (95% CI)
**Intention to vaccinate before GP visit**						
Bad/good	2.670	0.698	14.609	1	0.000	14.437 (3.672-56.758)
GP doesn't support/support	0.524	0.309	2.873	1	0.090	1.688 (0.921-3.094)
Constant	-16.699	4.688	12.686	1	0.000	0.000
**Vaccination with aTIV**						
Age (years)	0.169	0.082	4.253	1	0.039	1.185 (1.008-1.391)
Foolish/wise	1.652	0.389	18.018	1	0.000	5.219 (2.434-11.193)
Constant	-19.790	7.252	7.446	1	0.006	0.000

aTIV, adjuvanted trivalent influenza vaccine; B, coefficient for the constant; df, degrees of freedom; GP, general practitioner (health care provider or physician); SE, standard error; Sig., significance. [1]Variable(s) entered in initial model: age (years), education level, income level, bad/good, foolish/wise, ineffective/effective, dangerous/safe, unimportant/important, important people don't support/support, GP doesn't support/supports. Final model presented upon backward selection using the Wald method.

Table S3. Responses to open-ended patient questions answered prior to a visit with healthcare provider/physician (each row represents a different patient's response).

What do you know about seasonal flu infection?	What do you know about seasonal flu vaccination?	Last winter, did you get a seasonal flu vaccine?	Why?	This current winter, will you get a seasonal flu vaccine?	Why?
Nothing	No	YES	Because it's a good thing. Offered something that can help me	YES	[Nothing written]
During winter flu is common and in vulnerable people it can be fatal	Covers some of the flu viruses likely to be circulating	YES	Flu prevention	YES	Flu prevention
Can make you very ill	A difference in age vaccine	YES	Because I take immunosuppressants	YES	I'm old and ill
Only symptoms	No	YES	[Nothing written]	YES	[Nothing written]
Dangerous to elderly/ill/previous probs children	No	YES	[Nothing written]	YES	[Nothing written]
No	No	YES	Important	YES	[Nothing written]
No	No	YES	Because I was sent for	YES	[Nothing written]
Not good for older/young people	Apparently works	YES	[Nothing written]	YES	Apparently works
Feverish. Aches and pains. Different from a cold	No	YES	Sensible	YES	[Nothing written]
A one-off strain	Specific for strain of flu	YES	Flu is dangerous over '65'	YES	I don't want to get flu
Nothing	It is given to a certain group of people who need extra help to avoid getting the flu	YES	Advised by practice nurse	YES	To give me the best chance of avoiding flu
If you are not healthy it can be very detrimental to your health and even kill	Nothing!	YES	My husband is disabled after a stroke and he strongly advised me to take it and my GP	YES	Because I love and respect my husband and family. I need to be healthy to look after them
Good for senior adults. Less likely to get infections	[Nothing written]	YES	Scheduled	YES	Well managed by surgery
Not good for you if not	Prevent flu infection	YES	[Nothing written]	YES	[Nothing written]
Each year NHS and others select what they think will be the most likely (dominant) flu strain and select vaccines	This is the selected vaccine for the dominant flu strain	YES	To avoid catching flu	YES	To stop catching flu

What do you know about seasonal flu infection?	What do you know about seasonal flu vaccination?	Last winter, did you get a seasonal flu vaccine?	Why?	This current winter, will you get a seasonal flu vaccine?	Why?
appropriate for its treatment					
Not a lot	Not a lot	YES	Doctors advise	YES	Doctors advise
Can affect patients' health badly	Helps to avoid complications from flu infection	YES	Good for you	YES	Good for you
Can be dangerous to people with diabetes	Protects against flu infection	YES	[Nothing written]	YES	[Nothing written]
[Nothing written]	[Nothing written]	YES	Recommended by practice	YES	[Nothing written]
[Nothing written]	[Nothing written]	YES	[Nothing written]	YES	[Nothing written]
Helps to combat the flu could help to resist any other illness	Helps to combat the flu could help to resist any other illness	YES	Flue can be fatal [illegible] in the elderly	YES	Flue can be fatal[illegible] in the elderly
Can be dangerous if not done to older people	Older people can feel the benefit	YES	Health wise	YES	I feel the benefit
Annual occurrence	Necessary	YES	[Nothing written]	YES	[Nothing written]
Flu vac only effective against target strain - I have suffered from a flu- like virus this winter	Harmless and effective against selected strains of flu	YES	Flu is a very unpleasant, sometimes dangerous infection therefore avoid if possible	YES	I have had pneumonia twice in one year
Old people are more vulnerable	Doctor gives it to old people	YES	[Nothing written]	YES	[Nothing written]
Makes one feel miserable and lethargic	Wards off the infection or at least minimizes the risk	YES	Doctor recommends annual flu shot. Have had shots for many years	YES	Wards off the infection or at least minimizes risk
An infectious disease of the respiratory system [illegible] occurring in winter	To protect against diseases during the winter season	YES	Common sense	YES	Prevent illness
Protects against getting full blown flu	Protects against getting full blown flu	YES	Protection against flu	YES	Protection against flu
It may kill me	To protect against flu and any other health issues I may have	YES	To protect me thru winter	YES	I feel safer having it as I need to keep well
Not a lot! Each season the flu infection varies slightly so making a vaccine against a	Vaccine is grown in egg [illegible] to be effective against a predicted type	YES	I think it has some effect	YES	I think it has some effect

What do you know about seasonal flu infection?	What do you know about seasonal flu vaccination?	Last winter, did you get a seasonal flu vaccine?	Why?	This current winter, will you get a seasonal flu vaccine?	Why?
predicted type is a little uncertain					
It sometimes gets bad press but myself and family have never had bad side effects	[Nothing written]	YES	Sensible to have it	YES	[Nothing written]
It can be very debilitating	Nothing other than the advice given by the doctor	YES	On the advice of my doctor	YES	I have had the 'flu shot for the last few years and have found it very helpful
Each year the flu strain changes and certain strains can kill especially the young and elderly so anything that can help can only be good	The WHO monitor all around the world to see which strain is most likely to be around culture the vaccine to make the best effective flu shot for that year	YES	I am diabetic and aged 70yrs and have been getting it yearly since I was first given it in about 2000	YES	Because in all the time that I have been getting the flu jab I have only had flu once and that wasn't too bad
Causes ill health	Prevents flu	YES	Prevents flu	YES	Prevents flu
Prevents some types	Prevents some types	YES	[Nothing written]	YES	[Nothing written]
It can be a killer	[Nothing written]	YES	Have it every year	YES	[Nothing written]
Most people get it but I don't think I've ever had it	It is supposed to stop you getting flu	YES	Because I was told at the surgery I should have it	Not specified	Not sure
[Nothing written]	[Nothing written]	YES	It was advised	YES	[Nothing written]
Can be dangerous to older people	Have been encouraged to have it	YES	Encouraged by GP	YES	Seems sensible to avoid flue if possible
[Nothing written]	That it is important to have	YES	[Nothing written]	YES	[Nothing written]
If you have the injection, your chances of getting flu is very low indeed	Nothing	YES	I am a Type 2 diabetic, I have had the flu jab at least 16 years	YES	I get sent an appointment from my doctors
It is extremely debilitating and the person becomes very ill	It only takes a few seconds	YES	To be prepared	YES	To be prepared
[Nothing written]	[Nothing written]	YES	To hopefully prevent me from getting flu	YES	To continue to help prevent me from getting flu
Very bad for elderly and or various health problems	Hopefully should protect against the worst outbreaks	YES	[illegible] with heart problems (have fid def pacemaker)	YES	[illegible] with heart problems (have fid def pacemaker)

What do you know about seasonal flu infection?	What do you know about seasonal flu vaccination?	Last winter, did you get a seasonal flu vaccine?	Why?	This current winter, will you get a seasonal flu vaccine?	Why?
That it helps if you get flu	Help me	YES	[Nothing written]	YES	I want to take extra care now
Seasonal flu is very infectious to others	It helps to reduce the chance of getting flu	YES	I am a great believer in all eligible patients getting a flu jab to reduce the incidence of annual flu	YES	I am a great believer in all eligible patients getting a flu jab to reduce the incidence of annual flu
It can be dangerous and lead to death	Don't know	YES	Just to be safe and stayaway from flu	YES	I do it every year
Very dangerous	Can be beneficial	YES	Because I was recommended to have it due to my diabetes	YES	Because I was recommended to have it due to my diabetes
Makes you very ill	Not very much	YES	Thing that you have to do, needed and wise thing to do	YES	Thing that you have to do, needed and wise thing to do
Very dangerous virus - kills children, old people	Nothing	YES	I've had flu previously	YES	I want to stay alive
I don't know a lot. I just have my flu vaccine and hope it sees me through the winter	I don't know anything about it	YES	Because it's natural to get it	YES	Because it's natural to get it
Not a lot to be quite honest	They change the flu vaccine every year	YES	So you didn't get colds or flu etc	YES	Because all the others have worked
It can cause you to go to bed and out of action for 3-4 weeks	Different strains in it every year	YES	Because I have one every year	YES	Because I've always had them
Complicated issue. If it is prevalent it is wise to have protection if you can	Research goes into what strain is prevalent - it mutates into different strains	YES	Hopefully to remain moderately immune	YES	To offer me reasonable protection
Nothing	What I have read in the press	YES	I thought it was the best thing to do	YES	[Nothing written]
Can be deadly/ minimise your risk as much as possible	Very little but I thought it covered one strain of flu	YES	Hopefully helpful in not getting flu	YES	Hopefully helpful in not getting flu
Usually have a very high temperature, nose runs like a tap, can turn to pneumonia I believe	Nothing really	NO	Because I didn't want it and I don't normally get flu	NO	Because I didn't want it and I don't normally get flu

**What do you know about seasonal flu infection?**	**What do you know about seasonal flu vaccination?**	**Last winter, did you get a seasonal flu vaccine?**	**Why?**	**This current winter, will you get a seasonal flu vaccine?**	**Why?**
It can be horrible	Researchers take what they believed to have caused flu in the previous year and produce a vaccine to stop it	YES	To build up resistance to those strains of flu	YES	To build up resistance to those strains of flu
Debilitating - especially at age 68, I am a childminder I can't afford to have flu	Prevent you getting flu	YES	Because I have a lot of common sense and thought it was necessary	YES	Because I have a lot of common sense and thought it was necessary
Puts you on your back	It has something added to it. I have had no repercussions having it, worthwhile having it	YES	I have a heart condition, vital for me to have the flu vaccine	YES	I have a heart condition, vital for me to have the flu vaccine
Horrible to get - not something you want, confined to bed, worse as you get older, bad for young children	Best to take the vaccination	YES	Sensible thing to do	YES	Better than getting flu
Gives you a temperature - feel lousy	They pick a vaccine that they feel is effective for that year	YES	As a precaution because I have an asthmatic chest	YES	As a precaution because I have an asthmatic chest
Can be quite dangerous	Nothing	YES	Because it was available and it was for my benefit to have it	YES	I intend to have the vaccine because I feel it protects me from the flu which can make me quite ill
It's around everywhere - affects young and old. Old people have not got a good immune system	It's there for a reason - to do the job it's supposed to do	NO	Previous bad reaction the year before when I had 2 injections together	YES	You have to build your immune system up
Very debilitating - can be dangerous to certain people	Different each year	YES	I have asthma and I am 'of an age'	YES	I have asthma and I am 'of an age'
Alters every year	Dead virus. Vaccine alters every year	YES	Nurse told me to have it and gave it to me	YES	So I don't get flu

**What do you know about seasonal flu infection?**	**What do you know about seasonal flu vaccination?**	**Last winter, did you get a seasonal flu vaccine?**	**Why?**	**This current winter, will you get a seasonal flu vaccine?**	**Why?**
Changes every year	Not a lot apart from it works and it changes every year	YES	Because I always have it	YES	Because I don't want to get flu
[Nothing written]	Nothing	YES	Offered it because of my age	YES	To prevent me from getting flu
Can make people poorly with a high temperature and can't get out of bed	Offered to people with certain illnesses and people over 65 pregnant ladies	YES	Because I was offered it	YES	Prevent me from getting flu
I have had flu twice; I was ill and bedbound	It suits me I haven't had flu since being vaccinated	YES	Because I don't wantful	YES	Because I don't want flu
Every year can be a different strain	Vaccines are developed to try and protect against this season's strains	YES	Advised to	YES	Advised to and sensible
Effects older people and younger children	Very effective	YES	First year has met NHS criteria	YES	Very important
It can vary throughout the world	Nothing	YES	Advised by GP and hospital doctors	YES	Because it has worked for me
[Nothing written]	Better to have than not	YES	Don't want to get flu	YES	Don't want to get flu
Nothing	Nothing	YES	Advised to by doctor	YES	Advised to
Can cause aching joints and feel unwell	Protects against flu	YES	To prevent flu	YES	To prevent flu
There are different strains, result in ill health. Can be fatal in the elderly	Doesn't always cover all strains of flu	YES	Advertised	YES	Important
Seasonal occurs autumn and winter. Very uncomfortable. Can stop usual activities	If you get a cold it is not as bad if you have had the vaccine	YES	Can't afford to be unwell with other current health problems	YES	Can't afford to be unwell with other current health problems
More prevalent in winter months particularly around xmas when people are mixing. Can be very dangerous particularly for people with lung and heart problems	Aimed on what flu is thought to be the strain circulating in the coming season. Available from mid September	YES	Always get the vaccine	YES	Because believe in having all vaccinations. Prevention is better than cure

What do you know about seasonal flu infection?	What do you know about seasonal flu vaccination?	Last winter, did you get a seasonal flu vaccine?	Why?	This current winter, will you get a seasonal flu vaccine?	Why?
Seasonal only occurs in winter. Increased exposure in crowded areas. At increased risk from grandchildren. There can be milder variations of flu, different strains	Painless. Flu vaccine doesn't completely stop people from getting flu but reduces the risk	YES	In the habit of getting flu vaccine. Wife encourages	YES	Free
Very little	Possible side effects, read about it in the paper	YES	Always had it	YES	Always had it
It can make you very ill	Quick, easily administered	YES	Always had it. Had to have it when I was a nurse	YES	I just will, it's good for me
Nothing	Nothing	NO	Had once and had a bad reaction	NO	[Nothing written]
Nothing	Nothing	YES	Kidney disease	YES	Important for health. Work at a swimming pool, hot environment, more likely to get ill
Nothing	Nothing	NO	Didn't think it was necessary. Not keen on needles	NO	Fear of needles
Shift of strains, vaccine is adjusted accordingly. Based on eggs	Shift of strains, vaccine is adjusted accordingly. Based on eggs	YES	Advised by asthma nurse	NO	Because I had whooping cough vaccine
Nothing	Nothing	YES	Because I was told to	YES	Because I was told to
Different strains. Read that it's better to get it in the morning than afternoon	[Nothing written]	YES	Had correspondence from surgery	YES	Always had it
No	What I read	YES	To try to not get the flu	YES	Not to get flu
High temperature, colds, makes you poorly, bed bound	It's well advertised to have it every year. On noticeboard, in paper	YES	Prevention	YES	Prevention, better to have it as getting older
Spreads about, touching handles	Not a lot. Different strains each year, side effects	YES	[Nothing written]	YES	Don't want to get pneumonia
Lays you up for a month. You know	Contains 2 flu strains	YES	Important - I don't want flu	YES	I don't want to get poorly

What do you know about seasonal flu infection?	What do you know about seasonal flu vaccination?	Last winter, did you get a seasonal flu vaccine?	Why?	This current winter, will you get a seasonal flu vaccine?	Why?
when you've had flu					
Very little	Very little	YES	Always had it. Daughter is an HCP so encouraged me to have	YES	Always had it. Daughter is an HCP so encouraged me to have
Nasty - lay you down for about a month	Nothing much really. Grown on egg embryo	YES	Reduce risk of flu	YES	[Nothing written]
Contagious, uncomfortable, nuisance	Side effects are okay, last a day. Worthwhile having the vaccine	YES	Anticipation of flu - don't want it	YES	I don't want flu - want to reduce my risk
Spread by coughs and sneezes	It's a vaccine. Safe	YES	I have asthma, COPD and a heart condition	YES	Because of my health
Not a nice disease, not something you want to have	Not much about	YES	Stop getting flu	YES	Stops me getting flu
Debilitating. Can make you weak, not eat anything. Makes you feel awful, headache, worried, poorly for 2 weeks	Different every year, differs with age. Can be difficult to know what strains	YES	Don't want flu	YES	Don't want flu
Different strains	Different vaccines for different age groups	YES	[Nothing written]	YES	[Nothing written]
No	Had for 30 years and not had the virus, so very effective	YES	[Nothing written]	YES	[Nothing written]
Nothing	Nothing	YES	[Nothing written]	YES	[Nothing written]
Very little	Very little	YES	[Nothing written]	YES	[Nothing written]
Can be extremely serious	Should/can help avoid suffering from flu	YES	Hopefully to avoid flu symptoms. Helps to keep other people/ myself from catching flu	YES	Hopefully to avoid flu symptoms. Helps to keep other people/ myself from catching flu
To experience seasonal flu infection could have serious consequence including possible death, particularly among the elderly	I accept and trust flu injection given	YES	[Nothing written]	YES	[Nothing written]
Very serious for older people (can be)	It seems to work	YES	I agree with having vaccinations when no ill effects with past one	YES	I agree with having vaccinations when

What do you know about seasonal flu infection?	What do you know about seasonal flu vaccination?	Last winter, did you get a seasonal flu vaccine?	Why?	This current winter, will you get a seasonal flu vaccine?	Why?
					no ill effects with past one
Different strains	Different choice of flu vaccines and surgery choice best option for>65 yrs	YES	To protect myself	YES	To protect myself
N/A	N/A	YES	Choice	YES	Choice
Very little	Very little	YES	[Nothing written]	YES	[Nothing written]
It effects anyone at any age. 2 difference vaccines this year unless 65 yrs and older than 65 yrs	I got the best vaccine for my age this year	YES	To protect myself from flue. My GP invited me in to receive it	YES	To protect myself and my family
Not a lot, only from media, other people's experience. But it can be dangerous, especially for elderly or very young people	It provides resistance against some but not all flu viruses	YES	[Nothing written]	YES	[Nothing written]
Nothing	Improves chances of avoiding flu virus	YES	[Nothing written]	YES	[Nothing written]
Not a great deal	Only what the nurse tells me. 3 different vaccines, one [illegible] for <18, one for <65yrs and one for >65yrs	YES	[Nothing written]	YES	[Nothing written]
It is very dangerous and debilitating and can in some people cause death	It covers the strains that are 'most likely' to cause problems this season and it makes sense to receive it!	NO	I was not 65	YES	Because I don't want to contract flu and my GP advises to be vaccinated
Can be devastating, some people who have the jab get ill but thankfully that has never happened	You can't have it if you have a cold. If I forget I should have it ASAP	YES	Always had it - wouldn't stop	YES	[Nothing written]
Can be very bad, can be debilitating. Can affect my chest because of my asthma	Gives me a little bit of the flu to make my body immune to it	YES	To avoid having the flu, worried a free winter	YES	[Nothing written]
Pretty devastating. No one knows what the flu is like	Should have it yearly	YES	Prevent getting unwell	YES	[Nothing written]

What do you know about seasonal flu infection?	What do you know about seasonal flu vaccination?	Last winter, did you get a seasonal flu vaccine?	Why?	This current winter, will you get a seasonal flu vaccine?	Why?
until they have had it					
Can be a killer	Can have side effects -protection side is greater	YES	To keep well	YES	To keep well
Flu is really horrible!	Just a jab and done	YES	To keep well	YES	[Nothing written]
Makes people very unwell and very quickly	Need to have yearly	YES	To protect myself and my family	YES	[Nothing written]
Not very nice, shivering, temp, cough, etc	Helps to safeguard me	YES	To help me	YES	[Nothing written]
Can lead to pneumonia and sickness	Helps me not have problems. Have to have every year	YES	To avoid sickness	YES	To avoid getting unwell
Do not know anything	Every year, will get a red sore spot on arm	YES	Because it is sensible	YES	[Nothing written]
Can range from mild to very severe with life threatening results. Can mutate year to year its form	Not 100%, can't be. Varies year to year in effectiveness. I would rate that better than getting flu. Some groups are against vaccines not just flu one. I rate this an[illegible]	YES	As always. Protection against flu realise not 100% but flu can be more serious than people think	YES	As always. Protection against flu realise not 100% but flu can be more serious than people think
Nothing - can be very serious. Changes form every year, different flu germs	Meant to be dead vaccine can't get flu from it	YES	Doctor recommended	YES	Doctors recommended
Can lead to death, especially in older people and young children. It's important that all relevant ages have the vaccine. Can even make healthy people feel very unwell	Has to be planned and vaccine based on the information they have ready for the next year. Sometimes it isn't quite right for the flu that's around that year	YES	Need to keep healthy as I live alone	YES	Keeping well. I live alone so very important
Had it once - you would never confuse flu with a cold. When you have had it, it really knocks me out	Thick it is pretty effective not 100%. Urban myth that it gives you flu but I don't believe that some people say they have had	YES	If I am called from my GP I obey	YES	If I am called from my GP I obey

What do you know about seasonal flu infection?	What do you know about seasonal flu vaccination?	Last winter, did you get a seasonal flu vaccine?	Why?	This current winter, will you get a seasonal flu vaccine?	Why?
	worse flu from getting it				
Changes every year. Can be very dangerous for older people or people with certain problem COPD etc. Also young people with underdeveloped immune system. I'm not especially at risk but important to be vaccinated	Recommended that people over 65yrs get it. Its optional - can get from chemist if not doctor. I did the last 2 years. Do not give to people with egg allergies. Some people can have bad reactions	YES	Planning to have it every year as over 65yrs. Helps to prevent population not just me from getting flu	YES	Planning to have it every year as over 65yrs. Helps to prevent population not just me from getting flu
Had it once - won't want it again, worried be worse at my age	Based on eggs. Can get a reaction to the vaccine	YES	Have it every year	YES	Have it every year
Symptom you get - aches and pains feeling really ill	What I have heard, I am told injected with a strain of flu	NO	I consider myself for my age really fit and able to cope. Some elderly people may need it	NO	Same reasons, think I could fight it off
I have had it so know it is nasty. Very infectious and debilitating. Good there is a vaccine that can stop you getting it	Know it changed last year for older people to stimulate the immune system. Easy to have, its simple, it's never affected me badly	YES	It's necessary to have it if offered	YES	Same answer - don't want flu
Around for years and years and there are different types of flu every year. Dependent on one's own health can make you more prone to flu	Only thing I know that they are constantly trying to improve it so different vaccines every year	YES	Had it for a least 15yrs and its worked 90% of the time. I have not had flu despite being chesty (asthmatic chest) but not in summer month	YES	Always worked in the past. I am getting older and getting chestier
Affects me badly. Flu puts me on my back. My lungs very bad so don't want to catch flu	Prevents me catching flu, why not have it?	YES	[Nothing written]	YES	[Nothing written]
Varies from mild to deadly. Some of my family died of flu in 1918. As you get older less able	It's targeted each year to what they think the flu will be this winter, bit hit and miss but	YES	For all above reasons, it's a good idea, severe [illegible] people say it helps against colds and	YES	For all above reasons, it's a good idea, severe [illegible] people say it helps

What do you know about seasonal flu infection?	What do you know about seasonal flu vaccination?	Last winter, did you get a seasonal flu vaccine?	Why?	This current winter, will you get a seasonal flu vaccine?	Why?
to fight it off. You can die from it especially if frail or elderly	good at doing its job. Varies from year to year in effectiveness		other infections although not proven - yes it's worth it		against colds and other infections although not proven - yes it's worth it
Knocked me off my feet 7- 10 day, high temp, chest poorly, generally unwell, aches and pains	Not a lot. Something to do with chickens, some people say it doesn't work	YES	[Nothing written]	YES	[Nothing written]
Puts you in bed, make you weak, leaves you with complication - chest etc. Not a nice thing to have, avoid if feasible as comes every year	Doctor tells me when due and says you need it that's all I know. Get a letter saying flu vaccination is due	YES	Don't want flu. Had flu 4-5 yrs ago and never since	YES	Same reasons but also as you get older get more complications so need to keep healthy
I have had it and very different to a cold. Body aches, all over, skin tender, dry hard cough	No nothing	YES	Always have it. Offered when [illegible] did have to come back	YES	Because I have it every winter
It's debilitating, puts you on your back if real flu. Takes a while to get over, not like 'man flu'	I get it in my arm and that's it!	YES	Because I get bronchitis	YES	Because I get bronchitis
Very debilitating, people die from it, dangerous for asthmatics (my husband). Never had it but witness it	I don't know anything apart from I [illegible] and have it. Know here is a new one this year but I haven't noticed any differences	YES	That it's necessary as it's a preventive measure, don't want to have flu	YES	That it's necessary as it's a preventive measure, don't want to have flu
Like a heavy cold, you feel weak and tired. Takes 4-5 days to get over it, you would neurophen to treat it	Not a lot, been around a while, lots of people have it, some say it works some say it doesn't work, you can get a cold with it	NO	Don't think about flu so won't bother	NO	Don't think about flu so won't bother
Not much at all but highly infectious. Can be rather dangerous to people with other ailments or older people	Nothing	YES	Have had it every year- routine	YES	It might stop me catching the flu. I am getting older so would be more effected by flu

What do you know about seasonal flu infection?	What do you know about seasonal flu vaccination?	Last winter, did you get a seasonal flu vaccine?	Why?	This current winter, will you get a seasonal flu vaccine?	Why?
Nothing. Fevers, every year could get bronchitis but vaccine has helped. Can't move, can't work, can't get out of bed. Weakness of limb	Nothing - imagine it gives you a dose of flu	YES	It's been effective in past years. Helps me not need antibodies for my COPD/bronchitis	YES	What to avoid bronchitis and as above
It's dangerous, gives you sneezes and colds	No real opinion about it	NO	Can't remember why	YES	[Nothing written]
Can cause pneumonia. Lack of energy, can't eat or drink. Leads to other things. High temperature	Just had it when I came in, didn't notice anything. Had flu afterwards so not convinced	YES	Cause doctor said to have it	YES	Not sure, had flu anyway
Comes in many forms, can change every season. Killed a lot of people - Spanish flu- filled more than WWI	I understand it to be a watered down type of flu. Gives my body something to fight against	YES	Because I have had it for last 4-5yrs	YES	Because I have had it for last 4-5yrs
Not very much other than it can be dangerous and you can die from it, high temp, etc	Nothing about that, it's probably a dose of the flu so effects immune system	YES	Important to do so	YES	Important to do so
People who get it could be very ill	Don't know	YES	Because I have not had flu since having it	YES	Because it insensible
Very disabling, leading to chest problems	Prevents flue	YES	For protection against flu	YES	Again, for my protection against flu
Not a good thing to have!	Mixed vaccine	YES	Protection from flue	YES	Protection from flu
I was recommended in 2005 to have the flu jab following triple by-pass heart surgery	It is [illegible] to control flu infection	YES	[Nothing written]	YES	[Nothing written]
Is proven to be effective	[Nothing written]	YES	I intend to have it every year	YES	[Nothing written]
Not a lot other than difficulty in covering all strains	Nothing	YES	Guidance by nurse	YES	[Nothing written]
The vaccine for over 65's has changed this year. Flu can't be cured	The vaccine for over 65's is new this year	YES	Was encouraged by practice	YES	Own protection. Less of a burden on third parties

What do you know about seasonal flu infection?	What do you know about seasonal flu vaccination?	Last winter, did you get a seasonal flu vaccine?	Why?	This current winter, will you get a seasonal flu vaccine?	Why?
Flu can be a serious infection particularly for the elderly or those with chest problems e.g., asthma	Carries 3 or 4 strains of the flu virus which the experts think will be most prevalent	YES	I get it every year	YES	It's important and I get it every year
[illegible] in hospital if I did not have it	Made up of different strains	YES	[Nothing written]	YES	[Nothing written]
Very infectious, extremely uncomfortable, can be life threatening	Hopefully shields you against catching it and passing it on to others	YES	Because I thought it was the sensible thing to do - as it was available	YES	Because I thought it was the sensible thing to do - as it was available
They occur	Sometimes not as successful	YES	Sensible	YES	Sensible
Vaccine varies from year to year so needs new vaccine every year	More or less painless, must be done every year	YES	[Nothing written]	YES	[Nothing written]
Can be caught it different ways, avoid people who have flu	Each year it different strain, and tested	YES	[Nothing written]	YES	[Nothing written]
It varies from year to year. It may be detrimental to the health of the elderly like me, and can cause death	It is adjusted to cope with new strains of the virus in order to prevent as far as possible people catching the disease. A great deal of research is carried out to that end	YES	I felt the advice from the surgery to have a shot was sensible. I trust my doctors; I did not want to catch flu if possible	YES	It seems a good idea as I have not had flue for several years - probably because of the flu vaccination
I have become more aware of the 'seasonality' in the past 20 years and recognise that it can be very debilitating	I understand there is a need to predict the likely flu strain in advance of its arrival and that there is a longish lead time in producing effective vaccines	YES	Advised by GP	YES	Prevention is better than cure
Very little	Not uncomfortable	YES	Provided by surgery	YES	Helps avoiding flu
Becomes more prevalent after start of school term. Varies in type from one year to the next	It is not a live vaccine which is important to me	YES	It has worked effectively in previous years	YES	Previous effectiveness

What do you know about seasonal flu infection?	What do you know about seasonal flu vaccination?	Last winter, did you get a seasonal flu vaccine?	Why?	This current winter, will you get a seasonal flu vaccine?	Why?
Recommended by others. Part age related	Available, FOC on demand	NO	[Nothing written]	YES	[Nothing written]
There are a variety of strains [illegible] not guaranteed to be the right one but mainly effective in stopping flu [illegible]	It's pretty [illegible] advised by medical professionals especially at risk patients such as myself	YES	Because I was advised [illegible] it was sensible	YES	I am an [illegible]!
Very important	Good for me	YES	[Nothing written]	YES	[Nothing written]
Hot temp, makes you feel very ill, cannot get out of bed, headaches, shivers	Best guess	YES	Don't want flu	YES	[Nothing written]
Its best avoided at all costs. Flu can kill old people I believe!	We get the publicity from the surgery through text messages, and we can choose where to have the vaccine	YES	I believe it's an individual responsibility to avoid illness wherever possible	YES	[Nothing written]
High temp, shiver, ache, lasts 7-10 days, feel very unwell. Potential chest infection	Different for over 65yr olds. Different strains combined. Had a reaction to the 2018/2019 vaccine-felt unwell for 2 days	YES	To protect myself especially as I have a lung condition	YES	To protect myself especially as I have a lung condition
Very little other than effects of flu can be most debilitating, consequently quite dangerous	Recommended by GP to prevent or reduce possibility of contracting flu	YES	[Nothing written]	YES	[Nothing written]
Severe infection making me very ill	A combination of 2 strains of flu virus - which WHO have recommended for that year	YES	I always have one	YES	[Nothing written]
High temperature, stay in bed	5 strains in vaccine	YES	Offered	YES	When offered
Bedridden, unable to cope	Combination of expected strains for that year	YES	Was a carer and needed it to protect myself and others	YES	I'm prone to flu round about March time so need protection
Very serious, bedridden, extremely ill for days, 4-5days	I know I will always have it. Feel it is important and	YES	Because I don't want to get flu	YES	Because I don't want to get flu

What do you know about seasonal flu infection?	What do you know about seasonal flu vaccination?	Last winter, did you get a seasonal flu vaccine?	Why?	This current winter, will you get a seasonal flu vaccine?	Why?
	helps herd immunity				
I know you can die of flu. Flu virus mutates. I know it is very different from cold. Serious illness	I know it changes every year. Sometimes get a reaction to it	YES	To protect myself	YES	To protect myself
Flu - very unwell for weeks. Different strains every year	Propagated in egg albumen. I find it painless	YES	I don't want flu	YES	My experience of flu was very unpleasant
Worse for people with other medical problems	Some side effects foursome people	NO	[Nothing written]	NO	Using homeopathy
Some seasons can be worse than others. Real 'flu can be serious and debilitating	It should protect me from the 'flu viruses	YES	I have it every year in the hope that it protects me	YES	I have it every year in the hope that it protects me
Very debilitating, headaches, sweating, fever- very ill, felt I was going to die	Need to have it before winter. Don't get it if you are unwell	YES	To protect myself, my family and others	YES	To protect myself, my family and others
It would be bad - could lead to chest infections etc	Very little	YES	I have asthma and have had a few chest infections during winter months and I was advised to have one	YES	Because of previous chest infections during winter months
It would be bad	I don't know	YES	To protect myself	YES	To protect myself
Symptoms get worse with age and if you suffer with any chest problems its very wise to have flu jab	Varying strengths and strains, changes every year	YES	Did it through work and therefore easy as on site - no travelling or waiting involved	Not specified	Unsure
Changes yearly. Unpleasant. Can be dangerous	Sometimes it is ineffective. Last year it seemed to work	YES	I have hyperthyroidism and atrial fibrillation and thought I would be more proactive than usual	YES	I have hyperthyroidism and atrial fibrillation and thought I would be more proactive than usual
Big difference between cold and flu. Bedridden. Don't eat. Chance of chest infection. No appetite. Very tired, very weak	Older people should have it. Think it is effective especially for 65yrs	YES	To protect myself	YES	To protect myself

What do you know about seasonal flu infection?	What do you know about seasonal flu vaccination?	Last winter, did you get a seasonal flu vaccine?	Why?	This current winter, will you get a seasonal flu vaccine?	Why?
I had it twice and it was one of the worst illnesses I have ever had. Makes you incapable, took a long to recover	It protects me from getting flu	YES	To protect myself and my partner	YES	To protect myself and my partner
Makes you very unwell. Terrible aches and pains, shivers. Bedridden. Worse than a cold	It changes every year. I trust that it works	YES	To protect myself	YES	To protect myself

Table S4. Responses to open-ended patient questions answered after visit with healthcare provider/physician (each row represents a different patient's response).

What do you know about seasonal flu infection?	What do you know about the adjuvanted seasonal flu vaccine?	What concerns do you have about getting the adjuvanted seasonal flu vaccine?	Some of the good things about getting the adjuvanted seasonal flu	Some of the bad things about getting the adjuvanted seasonal flu	Who would approve of you getting the adjuvanted seasonal flu vaccine?	Who would disapprove of you getting the adjuvanted seasonal flu vaccine?
More aware of infection and what it can do	Know its trivalent and for over 65's	No	Hopefully not getting flu	Some people have a reaction	All my family	None
[Nothing written]	[Nothing written]	[Nothing written]	[Nothing written]	[Nothing written]	[Nothing written]	[Nothing written]
[Nothing written]	Different vaccine	[Nothing written]	[Nothing written]	[Nothing written]	[Nothing written]	[Nothing written]
Fever, sore throat	3 strains	No	Prevention	No	Wife	No
[Nothing written]	Heightened vaccine for over 65's	No	Hopefully don't get flu. If you do, it reduces severity	Unwell for 3/52 with influenza. I always get a reaction every time	All groups	No
Nothing	Nothing	None	Protects me	No	Lives alone. No family	Lives alone. No family
Can affect older people worse	3 strains	No	Stops getting the flu	Don't know of any	Family	No
Very little	Very little	None	Hope it will stop getting the flu or to a lesser degree	None	Family	Nobody
Feverish, aches and pains	No	No	Be immune from flu	No	Family and friends	No
It helps to protect against flu	Specific strain	None	Protects against flu	N/A None	Friends	None
Nothing	It is given to avoid flu	None	Hopefully I will avoid getting the flu	Worried about possible side effects	Spouse, friends, work colleagues and management	None
It can be very debilitating	Nothing except it is used for over 65 generation	Don't know what it is and don't know what it does to my system!	I assume it keeps me safe	Made me feel flu like symptoms but one cannot tell if it is flu or a virus	My disabled husband and my family and friends as I am a full time carer for my husband	My daughter
Less likely to contract flu	Less likely to contract flu	None	Less likely to contract infections	[Nothing written]	Spouse/GP	[Nothing written]
Worse for older patients	Specific for over 65 years olds	None	Specific to older patients	None	All my friends and family	None

What do you know about seasonal flu infection?	What do you know about the adjuvanted seasonal flu vaccine?	What concerns do you have about getting the adjuvanted seasonal flu vaccine?	Some of the good things about getting the adjuvanted seasonal flu	Some of the bad things about getting the adjuvanted seasonal flu	Who would approve of you getting the adjuvanted seasonal flu vaccine?	Who would disapprove of you getting the adjuvanted seasonal flu vaccine?
There are various strains of flu that could be circulating in the winter and the NHS selects a vaccine appropriate for the most likely major one	Appropriate for the over 65's	None	Hopefully prevents one catching flu	Not known	Family and friends	None
Not a lot	Not a lot	None	Helps keep you well	None that I know of	Family and friends	None that I know of
Can affect older patients more	Better for older patients	None	Better for older patients	None	Family, friends and surgery	None
Can be dangerous to people with diabetes and older people	Recommended for older patients over 65	None	[Illegible] against more strains of flu	Reaction around injection area	Surgery, friends and family	None
[Nothing written]	More effective against flu	None	Prevents 'flu symptoms	None	Spouse, family	None
Bad for elderly	Specific for elderly	None	Protects from flu	None	Family and friends	None
Can be a killer	Designed especially for the elderly	None	Help to combat the flu virus	For myself, none	Age Concern	None
It was very helpful	The results are good	None	It helps people of my age	None	They all agree	None
[Nothing written]	[Nothing written]	[Nothing written]	[illegible]	None	Peers	None
An always unpleasant viral infection which can be serious for the elderly or young, or those with other health problems	A harmless flu vac that has been matched as well as possible to the current strain of 'flu	None	Peace of mind, hope that won't get flu or complications this season	It does not match all strains of 'flu	Spouse, family, medical practitioner	Some friends - "you always get flu if you have those vacs!"
Old people are in more danger	Doctor gives it out	None	Won't get flu	None	Family	No one
Any flu infection is debilitating but winter flu more so	Recommended to combat infection	None	Reduced risk, peace of mind	None	Family	None

What do you know about seasonal flu infection?	What do you know about the adjuvanted seasonal flu vaccine?	What concerns do you have about getting the adjuvanted seasonal flu vaccine?	Some of the good things about getting the adjuvanted seasonal flu	Some of the bad things about getting the adjuvanted seasonal flu	Who would approve of you getting the adjuvanted seasonal flu vaccine?	Who would disapprove of you getting the adjuvanted seasonal flu vaccine?
An infectious disease of the respiratory system	Increased protection in people aged 65 years and over	Mainly side effects - swelling/ inflammation at point of injection	Increased effectiveness in elderly people	Mainly concerned with [illegible]	Family/doctor	[Nothing written]
Protects against flu, harder to shake off when you get older	Protects against flu	None	Helps protect against flu	None	Family	Don't know
It could kill me	It protects against any health issues I may have	None	I feel safe within myself as I have other health issues	I may have a bad reaction but as yet this has not happened	Spouse, daughter and her family, friends	None
Seasonal flu strains are [illegible] that usually develop in the east and spread west, being most common in the winter in the western world	Which type of flu mutation is expected to spread is [illegible] vaccination. Strains are grown in egg embryos then the virus is [illegible] and killed to make it inactive, the body still reacts to produce antibodies to the deactivated virus	None	Can stop or lessen flu epidemics	Sometimes vaccine is ineffective against the strain of flu being transmitted	Friends and family	None
[Nothing written]	[Nothing written]	[Nothing written]	Avoiding flu	None	GP and medical team	[Nothing written]
I know that it can be very infectious	I know that it is necessary for the winter season and should be taken as advised	None	I found it quick and painless with very little after effects	None	Friends and family	[Nothing written]
The WHO monitor the flu situation around the world and decide which strain is going to be the most virulent and devise a vaccine to prevent infection	It is a vaccine cultured in the laboratory to effectively fight the relevant flu strain. I believe this year's was cultured using eggs, is	None	You don't get flu and if you do it is a mild dose	None	I would approve of young children, people with low immunity problems and the elderly, as well as people working in healthcare, getting it.	None

What do you know about seasonal flu infection?	What do you know about the adjuvanted seasonal flu vaccine?	What concerns do you have about getting the adjuvanted seasonal flu vaccine?	Some of the good things about getting the adjuvanted seasonal flu	Some of the bad things about getting the adjuvanted seasonal flu	Who would approve of you getting the adjuvanted seasonal flu vaccine?	Who would disapprove of you getting the adjuvanted seasonal flu vaccine?
	able to fight 4 different strains				Everyone would approve of me getting it	
Affects over 65s more	More effective for over 65s	None	Prevents flu and complications	None	Friends, family	None
Only prevents some virus's	Supposed to prevent more infection	At this time none	Prevents some virus	Cannot prevent all	Medical professionals	Don't know
It can affect elderly over 65	Helps over 65	[Nothing written]	Prevents flu	[Nothing written]	All	None
It is a very unpleasant illness	It boosts the immune system	It might in itself make me ill	[Nothing written]	[Nothing written]	[Nothing written]	[Nothing written]
It can be dangerous in older people	It is recommended to older people	None	Helps you fight the infection	None that I know of	Spouse, family	A few people have had bad effects from it
Real flu is not a cold. Needs to be treated with respect!	Can get from GP	None	Am 80: need to avoid flu	[Nothing written]	Spouse	[Nothing written]
Important to have	Nothing	None	Helps to prevent flu	[Nothing written]	Family	None
It makes you very ill	Not known	None	[Nothing written]	[Nothing written]	[Nothing written]	[Nothing written]
It is debilitating and the person is very ill	Don't know	None	It releases any tension regarding catching the 'flu	It hurts! Ha!	Husband and family	None
[Nothing written]	[Nothing written]	[Nothing written]	[Nothing written]	[Nothing written]	[Nothing written]	[Nothing written]
[Nothing written]	[Nothing written]	[Nothing written]	[Nothing written]	Sometimes get cold like symptoms	Spouse/friends etc	None
[Nothing written]	I know it is important to get flu vaccine as I have a weakness in my chest	I don't have concerns as I have had it for some time	I know it will help me if I get flu it won't affect as much	I don't know if there is any	Chest asthma patients, also Lucy (bad breathing)	Most of my friends all get flu jab
It is very infectious and has serious health complications for the vulnerable	It can help protect from flu	None	It may help reduce the incidence of flu in the elderly population	None	Spouse, friends, relatives, work colleagues	None
Can get poorly with it	Vaccine is to protect from getting flu	Side effect	Don't know	Don't know	Family, daughter, doctor	Son

What do you know about seasonal flu infection?	What do you know about the adjuvanted seasonal flu vaccine?	What concerns do you have about getting the adjuvanted seasonal flu vaccine?	Some of the good things about getting the adjuvanted seasonal flu	Some of the bad things about getting the adjuvanted seasonal flu	Who would approve of you getting the adjuvanted seasonal flu vaccine?	Who would disapprove of you getting the adjuvanted seasonal flu vaccine?
It can make you very unwell	Very beneficial for over 65's	No	Hopefully you are protected against flu	Can give you a sore arm	Health care professionals	Can't think of any
Makes you very ill	Don't know anything	No concerns - I have had no problems having the vaccine	Feeling of security	Possibility of side-effects	Wife, family	No
Happens more in the winter, dangerous, long recovery time	Developed for older people with added content	No concerns	It's free	I have never had any problems so no bad things for me	Family	None
Put to bed with it	Not a lot	No concerns	I get it and don't think about flu after my injection	No bad thing. It would be a bad thing if I didn't get it	Family	None
Not a thing	Not a thing	None whatsoever	I don't get any colds or flu right through winter	The older vaccines - my arm used to hurt for a week. The new one - my arm hasn't hurt at all	Family	I honestly don't know - I don't discuss it with anybody
Strike you down suddenly, in bed for 2-3 weeks	Don't know anything about it	No concerns	Prevent you from getting flu	Aggravation on arm	Family - son/ daughter	No one
Strain that becomes predominately prevalent at certain times of year	Different type/ combination this year	None whatsoever, I trust the medical professionals	Safeguards general health at a time of year where it is fairly essential	Can't think of any	Wife	None
Can be affected mildly or seriously	Don't know anything really	None - I trust the people who give it to me	Medical benefits	Not aware of any	Wife	Nobody
Can be deadly, minimise your risk as much as possible	Going to boost my immune system, contains 3 strains of flu, increase protection	Not going to cover 100% flu strains	Some protection - feel as if you have done something to help yourself against something horrid	Occasional soreness in your arm	Spouse, other members of family	No one I know
High temperature, wake up sweating	Added substance	I don't have the flu vaccine; I do know people who have had the flu vaccine who	To anybody who is susceptible to flu it's more preventative	No opinion	Friend	Can't think of anyone

What do you know about seasonal flu infection?	What do you know about the adjuvanted seasonal flu vaccine?	What concerns do you have about getting the adjuvanted seasonal flu vaccine?	Some of the good things about getting the adjuvanted seasonal flu	Some of the bad things about getting the adjuvanted seasonal flu	Who would approve of you getting the adjuvanted seasonal flu vaccine?	Who would disapprove of you getting the adjuvanted seasonal flu vaccine?
		have been worse than previous				
It can be horrible	Research based on the flu strains from the previous year	I don't have any	Help my immune system respond	None	Family	None
Debilitating at any age but to be avoided as a pensioner	Nothing	No concerns	Hopefully it keeps me from getting flu	None	Never discussed it with anybody	I can't think of anybody who would disapprove
Puts you on your back	Added substance	None whatsoever	Not getting flu	Only bad thing is not having the vaccine	Friends, family, spouse	Nobody
It's bad - not something you want	Best to have it	No concerns	Prevents you from getting flu and colds probably	Arm can be a bit stiff, don't feel like doing much afterwards	Friends, family	None
Cause high temperature, headaches, feel lousy	Prevent chest infections	No concerns	To prevent getting flu	I can't think of any	Wife and the staff/medical team at my surgery	I can't think of Any
Make you very ill and can kill people	Advisable to have it	No concerns as I have had no reactions	Easy to have it done and gives you peace of mind	I suppose some people can have a reaction	Friends and family	No
Cold and shivery, weakness	Different strain this year - added something to it	None - hopefully it works	Builds your immune system up	Don't want a reaction	Family/daughter in law	Idiots on the television!!
It can be dangerous and debilitating	Additive to help make the vaccine more effective	No concerns	If you do get flu it makes it a lighter flu than really bad	Sore arm	Not their concern - it's mine	It makes no difference to me - it's my decision
Changes yearly - alters its structure. Virus changes	Increases/helps immunity	None	Don't get flu - stay healthy	None - I've never had any problems	Family, doctor	None I can think of
I don't get it as I'm vaccinated against it	I don't know a lot	None really - it is all tested	I can enjoy life and go on my holidays	None	Friends	None - can't think of any
Makes people very unwell	Nothing	None	Prevents from getting flu	None	Family	None
Makes poorly high temperature and weak	Better for older people	None	Prevents from getting flu	None	Friends and family	None

What do you know about seasonal flu infection?	What do you know about the adjuvanted seasonal flu vaccine?	What concerns do you have about getting the adjuvanted seasonal flu vaccine?	Some of the good things about getting the adjuvanted seasonal flu	Some of the bad things about getting the adjuvanted seasonal flu	Who would approve of you getting the adjuvanted seasonal flu vaccine?	Who would disapprove of you getting the adjuvanted seasonal flu vaccine?
Can be painful, can cause people to become bedbound	Quite effective in my case	Hoping it will be effective	Prevents ill health	That it won't be effective	My children	None
Every year can be a different strain	Takes two weeks to be fully effective	None	Prevents people from getting flu	None	Spouse and children	Some friends believe they have had flu following the vaccine, but have caught a different virus
Effects older people	Different strains and more effective	None	Reduces the risk of getting flu and gives people peace of mind	None	Friends and colleagues	None
Can cause high temperature and bedbound for a period	Worked in my case	None	Prevents against getting flu	None	Spouse, doctors	None
Stops from getting flu	New strain because you can get immune to older strains	None	Hope I don't get flu	None	Family and friends	None
Can increase stroke risk	More important as you get older	None	Better response by stimulating immune system	None	Family	Some friends
Unwell, aching joints, can lead to pneumonia	Protects against flu	None	Makes feel more confident about going out	Nothing	Family and friends	None
Can cause pneumonia and be fatal in the elderly	Recommended to the over 65s. Helps to support the immune system	None	Strongly recommended by GP	None	Family	Some friends
Effects older people more than the younger	Effective. Don't get side effects personally	None	Less likely to cause flu	None	Family and healthcare provider	None
Seasonal Dec to March, at greater risk in crowded environments	Particularly important for over 65s as immune system	None	Helps protect against getting flu. The more people vaccinated the	None	Family	No

What do you know about seasonal flu infection?	What do you know about the adjuvanted seasonal flu vaccine?	What concerns do you have about getting the adjuvanted seasonal flu vaccine?	Some of the good things about getting the adjuvanted seasonal flu	Some of the bad things about getting the adjuvanted seasonal flu	Who would approve of you getting the adjuvanted seasonal flu vaccine?	Who would disapprove of you getting the adjuvanted seasonal flu vaccine?
	effectiveness decreases with age		reduction in the spread of flu			
Different strains. Seasonal in winter, increased risk to exposure in crowded areas ie. buses. Can make people very unwell	Different strains and different vaccines depending on people's immunity	None	More confident that no putting self at more risk	Misunderstanding that can cause flu but hasn't stopped me from getting vaccine	Spouse and family, daughter is a doctor	Some friends
It can be very dangerous, deadly	Better protection, specific for my age	None	It's a good thing to have	None	Family, friends	None
Ex nurse so aware of implications of flu	3 strains, dead virus, side effects	No concerns	Not getting the flu	Possible side effects	Doctors	Nobody
Helpful, comprehensive	I didn't know it existed until today	Don't want it, side effects	Can't think of any	Adverse reaction	Nobody has really mentioned it	Son and daughter in law
What HCP has just said	What HCP has just said	None	Peace of mind	None	Health professionals, daughter	None believers, spouse
Don't know	Nothing	Anti-needles. Never had flu	Prevent death. May stop me getting flu	Arm sore for 4 days, needles	Spouse and family	Probably nobody
[Nothing written]	It's different. 3 strains	No concerns	Not as ill if you have the flu vaccine	No comment	Medical, family, in general people	The moaners, people who are negative about it
Likely to be admitted to hospital. Less independent after having flu	Better vaccine for over 65. It's safe. Mild side effects	None	Not getting flu	None	Family	Nobody
Reduces the risk of flu	3 strains	None	Effective	None	Anyone except my wife	Spouse
Not pleasant. Have to take to your bed. Long time to get over it	Advertisement. What HCP tells me	None	Don't get flu	None, never had side effects	Family, HCPs	Daughters mother in law!
More healthcare needed after flu infection, increased risk of heart problems,	DN informed me, better, broader protection	None	Haven't had any flu symptoms since having flu vaccine	No effects	Family	Elderly - those that don't want it

What do you know about seasonal flu infection?	What do you know about the adjuvanted seasonal flu vaccine?	What concerns do you have about getting the adjuvanted seasonal flu vaccine?	Some of the good things about getting the adjuvanted seasonal flu	Some of the bad things about getting the adjuvanted seasonal flu	Who would approve of you getting the adjuvanted seasonal flu vaccine?	Who would disapprove of you getting the adjuvanted seasonal flu vaccine?
possible hospital admission						
3 strains. Dead strains	What I've been told, better protection	None	It's better for me, I have COPD so need it	Nothing really, I get side effects but they wear off in a few days	Doctor, family	Homeopathic believers
It can be nasty	3 strains. For over 65s	None	It works	None	Everyone	No one
3 strains. Dead strains	It's safe. It provides better protection	Not really	Reduced risk of flu	Nothing really	Family and friends	Homeopathic believers
It can have damaging implications if infected	It provides greater protection	None	Beneficial. Worth having	Nothing	Everyone	No one
It can be dangerous, life threatening. Could mean I'm in hospital	Better protection	None, a bit uncomfortable	Reduces flu risk, important	Uncomfortable	Family	Nobody, I wouldn't listen to anyone's opinion
Nothing really	Nothing, I will have it regardless	None, its important I have it	Effective	[Nothing written]	[Nothing written]	Nothing
Very ill, last thing you want if it can be avoided	3 strains. Effective, recommended	Nothing	Stops me getting flu	Not that I can think of. Not heard of anyone having an allergic reaction	Near relatives	No one
[Nothing written]	3 strains	None	Stop me getting flu	Nothing	Friends, never discussed with anyone	No one
If you have other conditions and >65 yrs it can be very serious and cause complications	Better injection to protect me from various strains of the flu virus	None	I believe/read that I only need to have this injection once in a lifetime	None	Spouse/family/ children	None
More likely in the winter	Not a lot. I understand the powers at be struggle to decide which strains to use to protect the population	None	Protection is great	None	Family and friends	None
NAD	Not interested. Trust GP and nurse	None	I am informed by my nurse that it is the best	None	None	None

What do you know about seasonal flu infection?	What do you know about the adjuvanted seasonal flu vaccine?	What concerns do you have about getting the adjuvanted seasonal flu vaccine?	Some of the good things about getting the adjuvanted seasonal flu	Some of the bad things about getting the adjuvanted seasonal flu	Who would approve of you getting the adjuvanted seasonal flu vaccine?	Who would disapprove of you getting the adjuvanted seasonal flu vaccine?
			vaccine available to protect me this year			
Not a thing	Not a thing	None	Stops the spread of flu	None	No input	None
Can be very serious. Can easily be passed to others	Should help to avoid catching flu therefore reduce number of sufferers	None	Peace of mind that hopefully I will be free of symptoms	? Any side effects	Spouse and children	None
Can have serious consequences including death, particularly among the elderly	Limited knowledge, but trust that it is beneficial	None	Additional protection for more strains of flu	None known	Spouse, children and friends	None
Can be very serious for older people	Supposed to be latest and best as it covers more types of flu	Hope that it is as effectives as the other one has been for me	[Nothing written]	[Nothing written]	[Nothing written]	[Nothing written]
Very little	Better for the older person aged >65 yrs	None	Extra protection if aged 65 or over	None	Family	None
Not a lot	Best vaccine for age group >65yrs	None	Reduce risk of getting virus so the health professionals tell me	None	Family and friends	None
Different strains	For the over 65s	None	It protects the >65's better	None	Friends and family	None
It effects anyone at any age	It's the best for >65yrs	No concerns	Protects me and my family	None	GP family - grandchildren	None
It can be dangerous for some groups of people, especially the elderly and very young	Provides resistance against some flu types	None	It's free. Little after effects. Provides some protection against flu infection, especially as I'm 69 years of age	I don't know any	Spouse/friends/ family/GP practice	I don't know of any
Nothing	Best protection for >65yrs	None	Best coverage	None	Family and friends	None
Various strains	Best vaccine for >65yrs	None	Best protection for my age group	None	None	None
The vaccine covers the strains of flu deemed	Nothing	No concerns at all	Hopefully protects me from contracting the	Can't think of any	Spouse	None

What do you know about seasonal flu infection?	What do you know about the adjuvanted seasonal flu vaccine?	What concerns do you have about getting the adjuvanted seasonal flu vaccine?	Some of the good things about getting the adjuvanted seasonal flu	Some of the bad things about getting the adjuvanted seasonal flu	Who would approve of you getting the adjuvanted seasonal flu vaccine?	Who would disapprove of you getting the adjuvanted seasonal flu vaccine?
to be a problem this season. Seasonal flu can be very dangerous for certain age groups and can be fatal			flu virus. It is painless and free			
Can be devastating	Boosts the immune system, as immune system weaker	None	Never get a cold!	No trouble with it	GP, nurse, husband	None
Flu can lead to death, runs you down for weeks. Makes you feel so weak and unwell	Combination of 3 strains of flu, injected with dead cells	None	Relief knowing I'm protected, takes the worry away because I'm vaccinated	None	Everybody around me	No
Devastating - you feel like you won't survive	Extra to boost my immune response to the vaccine	None	Protected	No	Family, friends	Have heard people say not too
It kills, makes you feel very very unwell, safeguarding me for the year	Contains something to boost immune - I accept the advice given to me	No I don't	Safeguards for the year, confidence that you are doing your best to stay healthy	Can have swelling and soreness	Friends, family - people who care about me	No
Is not pleasant	Know that I'm protected better	No	Peace of mind - I've done all I can	No	GP, family	No
[Nothing written]	Contains an ingredient to boost my response	None	Protects me, boosts my immune system	None	Family, GP, work	None
Not very nice	I know a lot more - it's really protection. I would not turn it down	Not now	Stops even colds	None	Family, friends	No - I don't listen to people
Makes you sick and unwell	Because I am older my immune system needs a boost	Not worried	I won't get ill	If it makes you feel unwell	Friends, pharmacist, doctors and nurses	None
Occurs every year	Contains oil to help my immune response	None	Protects me	Its individual responses	Daughter, doctor, nurses, friends	A friend

What do you know about seasonal flu infection?	What do you know about the adjuvanted seasonal flu vaccine?	What concerns do you have about getting the adjuvanted seasonal flu vaccine?	Some of the good things about getting the adjuvanted seasonal flu	Some of the bad things about getting the adjuvanted seasonal flu	Who would approve of you getting the adjuvanted seasonal flu vaccine?	Who would disapprove of you getting the adjuvanted seasonal flu vaccine?
Varies from mild symptoms to life changing symptoms. Knew about 1918 pandemic more than WWI people died. Mutates year by year various strains and vaccine changes to reflect that	Was not aware of the difference to other vaccine. Not aware that its booster additive was the extra bit	Very mild concerns - got risks but they are low especially compared to effects of flu if you get it	Less likely to get flu	They have a reaction. Something arm [illegible] ranging from minor to serious most would be minor	Family	None
Aware of symptom but reinforced that now. Importance of 65yr old to get best protection available	Not new vaccine. Know huge number doses given in Europe. Know it exists now	Following first treatment I had 4 cases of flu followed by sepsis. 6/12 off work and a very difficult period of recovery. Is this coincidence or related to vaccine?	Give immune system a boost	The uncertainty of not knowing if related to my flu experience. I have never been this poorly after a flu vaccine before. [Illegible] did not work. Including difficult every time in hospital treatment [illegible]	Immediate family.<br>Myself	None
Could lead to stroke and heart attack. Did not know 15,000 died in 2017. Did not know I had to have 2 weeks before effective	Adjuvant will carry it now to every cell in the body	None	More effective in over 65yrs	No. Some people say it makes them ill	Daughter and partner	None
Can kill a lot of old people, especially if other health condition. Did not know it could lead to heart attacks or strokes. Did know your immune system	Boosts the immune system makes vaccine more effective. It's never going to give you flu. Especially important if you have other health condition	None really	Slightly more effective for older people as immune system weakens. Help prevent complication such as stroke and heart problem	None	Husband, people I work and socialise with	None

What do you know about seasonal flu infection?	What do you know about the adjuvanted seasonal flu vaccine?	What concerns do you have about getting the adjuvanted seasonal flu vaccine?	Some of the good things about getting the adjuvanted seasonal flu	Some of the bad things about getting the adjuvanted seasonal flu	Who would approve of you getting the adjuvanted seasonal flu vaccine?	Who would disapprove of you getting the adjuvanted seasonal flu vaccine?
lessens as you get older						
Changes every year. Know how they decide on flu strains. Know had bad flu can be. It's not a bad cold - very debilitating	Designed to help over 65yrs. With age immune system deteriorates. Most people should have it unless allergies. I will continue to have it	With any vaccine there is always a concern about reactions but no more than normal	Peace of mind - less likely to get flu. Not passing onto others	Apart from some side effects no downside	Anyone who has thought it through. Friends, family	People who have a problem with vaccines
Need to have asthma and heart trouble etc can kill you	Did not know it was new vaccine	None	I didn't notice any difference. Should be more effective	No	Doctor, family	None. 1 friend doesn't have it - thinks it gives him flu
Same as before - makes you feel ill. If you have other illness it can make them worse	More effective than the older vaccine. Can [illegible] specific types of flu	Could get flu afterwards but this is probably wrong	Quicker actually, gives greater protection than previous vaccine	None that I can think of	Immediate family	None
Surprised by length of time it lasts thought lasts longer. Can make you depressed	Spread better around the body	No concerns	Seems to work better for older people	No don't think so	Family	None
It's a virus that can change so now come up with an added liquid that is added to normal vaccine, this will help people not get flu	Thought completely new vaccine, did not know had something added to normal vaccine to make it work better	No concerns, already had flu free year	Felt better this winter than last winter. Works well. Improvement good news	It's had 87 million doses so no bad things or they would have been seen by now	Wife, brother	No one
Worse than I thought. Can put you on your back. Proper flu terrible, makes you weak	Essential you get it, especially people with chest problem. Its more effective	None	Prevent me from being at risk of catching flu, keep me well	None	Family, friends, doctor	None

What do you know about seasonal flu infection?	What do you know about the adjuvanted seasonal flu vaccine?	What concerns do you have about getting the adjuvanted seasonal flu vaccine?	Some of the good things about getting the adjuvanted seasonal flu	Some of the bad things about getting the adjuvanted seasonal flu	Who would approve of you getting the adjuvanted seasonal flu vaccine?	Who would disapprove of you getting the adjuvanted seasonal flu vaccine?
Potentially dangerous, very expensive to NHS	Reasonable effectiveness at doing it jobs. More effective than previous model, spread around body better to increase effectiveness	Needle phobia, needle phobic	Decreases risk of getting flu	Not that I know of	Everybody I know, sister in law especially - she has paid for it for several years	None
Can be life threatening. Can cause extra heart problems	If successful hospital will have less elderly patient. That's all	None	Hopefully keep me fit, sounds promising	Nothing	Husband and mother	No one
Incapacitating plus carry risk of complication such as stroke or heart attacks. Lots of complication in the over 65yr immune system works less well	Very good idea should have had this years ago. Gives extra push round the system to make it work better	None it's been in Europe long enough. If any problem it would not be used	No problem, don't worry about the flu. I have had no effects, far as I know it's been ok	None	Family, friends in the nursing profession	People that don't like injections, don't believe it's good for your health
Killed a lot after WWI. Didn't that it was still a killer. Can exacerbate other condition i.e., COPD	Different flu vaccine, only for over 65yrs	None	Good if it prevents you getting flu	None	Doctors etc	None
Puts you on your back if real flu and not just a bad cold	Spread through the body quicker	No none	If it stops me getting flu that the best thing	No bad things	Family and friends	Sister something doesn't have it and she is not bothered, doesn't bother with vaccine
Learned that it can cause permanent debilitation especially in older people. Infection may last 1	Same as old vaccine but added adjuvant that spreads it around body getting it to all parts. Adjuvant is just an aid	None	Anything that improves function of vaccine must be commended	None - sometime arm may be sore but I didn't have that problem	Family, golf club friends, friends, neighbours	None

What do you know about seasonal flu infection?	What do you know about the adjuvanted seasonal flu vaccine?	What concerns do you have about getting the adjuvanted seasonal flu vaccine?	Some of the good things about getting the adjuvanted seasonal flu	Some of the bad things about getting the adjuvanted seasonal flu	Who would approve of you getting the adjuvanted seasonal flu vaccine?	Who would disapprove of you getting the adjuvanted seasonal flu vaccine?
week but recovery much longer						
How long since last outbreak, can kill you	Vaccine can save lives, advise others to have it I am still not sure myself	Will it affect my COPD and heart condition, will it affect me long term	New vaccine with extra chemicals, good use in Europe and they always need to improve vaccine	Will it affect my other health problems and medication so very wary to take it	Son	Some friends say not to have it
Now know many people with flu end up in hospital. Flu can have devastating effects	Feel the adjuvanted flu vaccine more effective than the usual flu vac	Only concern would be having shoulder problem following the flu vac given in 2017	Distributes it around the body in a more effective way	Don't know of any	Children, my son. Friends	None
More serious than I had previously thought. Not aware of the impact of flu on a range of health aspects e.g., Flu on top of diabetes or COPD, will make these worse	Takes account of WHO information. The delivery of the vaccine to bloodstream is enhanced by the adjuvant	Nothing	Most important it reassures me that I am protected from the impact of flu protection	Not aware of any	Everyone who care about me - family and close friends	Not at all in my circle
Nothing new from previous question	A lot better than old vaccine, better response to vaccine	No concerns	Not a lot different but sounds better	Nothing	None	None
Still got flu after having vaccine. What I said before basically	Helps it spread around the body, good for weaker immune systems	I react badly to some medication combinations	Back to last question - supposed to spread around body better	Something other than usual flu vaccine for body to cope with	Medical professional, family	None
Very debilitating and capable of killing you	It has a [illegible] in it to help elderly people pick it up better	I have no concern	For older people helps make sure I am getting benefits of the vaccine	Not that I am aware of	Family	No one
Can kill, effects over 65 more than younger ones	Extra, added constituent to flu vac	No concern	Extra protection if aged 65 or over	Not that I know of	Medical people in my family, my own GP	None

What do you know about seasonal flu infection?	What do you know about the adjuvanted seasonal flu vaccine?	What concerns do you have about getting the adjuvanted seasonal flu vaccine?	Some of the good things about getting the adjuvanted seasonal flu	Some of the bad things about getting the adjuvanted seasonal flu	Who would approve of you getting the adjuvanted seasonal flu vaccine?	Who would disapprove of you getting the adjuvanted seasonal flu vaccine?
	that keeps vaccine work more effectively					
Not a lot	Very effective	None	Prevents most types of flu	Sometimes a red area	Family	None
Two vaccines in one as extra protection. Oil enhances immune system. May have red mark at injection site	Designed for adults over 65 yrs	Nothing as it's not a live vaccine	Protect my immune systems	May have inflamed patch at injection site	People whose immune system is not good, elderly with chest problems	Certain groups who do not believe in vaccines
Very unpleasant can be dangerous	Natural oil added to enhance the immune system	None	No reaction to it	None	Family	None
It is a prevention measure to avoid flu infection	Did the job	None	To prevent clinical infection of flu	Natural reluctance	Family and friends	The negatives
[Nothing written]	[Nothing written]	None!	Not being extremely ill in the winter	Don't know of any!	Family and friends	None!
Not a lot	Nothing other than information provided by nurse	None	Hopefully to stay clear of infection	None known	Spouse, children and friends	None known
It is a new vaccine for over 65' and other vulnerable groups. It is not curable. Benefits gained by vaccination and good hygiene	It is a new vaccine that protects against 4 strains but especially effective with two in particular	None	Own health, less strain on the NHS	Can't think of any, apart from possible mild side effects	Spouse, family, NHS	Nil
Can be serious infection for elderly and those with chest problems e.g., asthma	Contains 4 strains of vaccine and natural oil to enhance immune system	None	Avoid getting flu	None	Family	No one who should be concerned about my health
Be in hospital if I did not have it	4 strains, natural oil to enhance my immune system	None	Stop me going to hospital and being very ill	None	Husband, family and health workers	None

What do you know about seasonal flu infection?	What do you know about the adjuvanted seasonal flu vaccine?	What concerns do you have about getting the adjuvanted seasonal flu vaccine?	Some of the good things about getting the adjuvanted seasonal flu	Some of the bad things about getting the adjuvanted seasonal flu	Who would approve of you getting the adjuvanted seasonal flu vaccine?	Who would disapprove of you getting the adjuvanted seasonal flu vaccine?
Very debilitating - knocks you out completely for at least 2 weeks	Apparently it has 4 strains which covers all aspects of the expected virus	None really - maybe a little discomfort from the actual injection	You don't contract flu!	Slight redness on my arm. Felt a little under the weather for a couple of weeks	Husband (definitely), children, mother	Nobody - I make my own decision
Occurs every year	Improvement on previous years	None	Free of flu	None so far	Spouse/friends	None
Can be spread easily so important to get flu jab	Not sure what this means. Now understand this makes it work better	None	Anything which prevents flu is great	No	All relations and friends	None I hope
Easily caught, get very poorly	Natural oil added to it, not a live vaccine	No	It might strain help me avoid having flu	None	None	Husband
It is an unpleasant disease to catch and may cause severe problems, even death, for the elderly	It is not a live vaccine and contains an oil to aid the effect i.e., to prevent catching various strains of flu	None - just a slight worry that it will hurt	It has stopped me getting flu	None really - except the slight discomfort of the injection and slight brief soreness	Family and husband if they bother to think about it	I can't think that anyone would worry about me be vaccinated to stop being ill
More aware now than in earlier days	That it protects against the most likely/common strains	None	Easily administered - in my case no discernable after effects	None	Spouse	None that I can identify
Very little	Very little	None	Hopefully do not get flu	Nothing	Family	No one to my knowledge
The flu infection tends to occur in early to late autumn and is transmitted via air droplets from sneezing or by physical contact. The seasonal flu can be particularly bad for the elderly	It is transferred towards the elderly. It is not a live vaccine	None having been assured it was not 'live'	Very simple to get, painless, and provided peace of mind. Did not have any reactions	None	Spouse and other family members	None

What do you know about seasonal flu infection?	What do you know about the adjuvanted seasonal flu vaccine?	What concerns do you have about getting the adjuvanted seasonal flu vaccine?	Some of the good things about getting the adjuvanted seasonal flu	Some of the bad things about getting the adjuvanted seasonal flu	Who would approve of you getting the adjuvanted seasonal flu vaccine?	Who would disapprove of you getting the adjuvanted seasonal flu vaccine?
Sensible thing to do considering age of 68	Designed for my age group	None	Reducing risk of flu	No known 'bad things'	Family and friends who have received flu vaccine	None
This year it targets at least 2 strains and it has kept me pretty much flu free all winter	It has this year got a natural oil in it which has to be good and left me with no reaction	None	Peace of mind, it has to be [illegible] medical history	It may not cover every strain of flu	Nurse, doctors, husband, some friends and pharmacist	A few sceptical friends
Make you poorly	Natural oil to make it work better	No concerns	Stop you from being ill	No bad things about it	Husband and family	Nobody disapproves
Makes you very ill, incapacitated, bed bound for long period	Helps over 65 dose more effective	None	No flu, less chances of getting flu	Sore arm	Older friends, family	None
Each year as I age it can be a worry as I have other symptoms that might be exaggerated	After this discussion I feel I know more about the reasons for the over 65 being given an adjuvanted vaccine	None	A feeling of confidence during the winter	None	Everybody I meet outside the home can be confident that I don't/won't spread the flu	None
High temp, shivers, aches, lasts 7-10 days, feel very unwell, potential chest infection	Special additive for over 65yrs so we absorb it better. To give better protection	No	Peace of mind. Feel I have done all I can to avoid flu and reduce pressure on NHS	No	Family, grandchildren	None
Know that it occurs and can have a variety of types (strains), can be very debilitating	Not a HVE vaccine, safe, limited physical impact	Only to know that vaccine is relevant to anticipated strain of flu (minor concern)	Possible protection from flu, comfort of knowing that I have done all I can do	None (in my case)	Medical profession, family (wife especially)	None
Nasty infection, most prevalent in winter months	Over 65 version	None	Hopefully, one remains healthy	None	Family and friends	None
What I read in media	Designed for 65+	No	Peace of mind	Not sure	Family	Not sure
Much more than a cold - prevents normal everyday life activity -	This gets absorbs quicker in elderly people so helpful	None	Hopefully will be more effective	None	Family	None

What do you know about seasonal flu infection?	What do you know about the adjuvanted seasonal flu vaccine?	What concerns do you have about getting the adjuvanted seasonal flu vaccine?	Some of the good things about getting the adjuvanted seasonal flu	Some of the bad things about getting the adjuvanted seasonal flu	Who would approve of you getting the adjuvanted seasonal flu vaccine?	Who would disapprove of you getting the adjuvanted seasonal flu vaccine?
become weak and bedridden with temp etc						
Very serious illness. Grandfather died of it	Specially designed for the over 65yrs	No	Stops me getting flu	No	Anybody with any sense	People who may have had a reaction
Serious illness	Specially designed for over 65s to increase immunity	None	Anything to reduce incidence for getting flu is a good thing	No	Anybody with sense	IDIOTS
A very unpleasant experience	It contains an additive to assist better take up in older people	None - so far!	Hopefully provides better protection than other vaccines	Unaware of any as yet	Family, friends	None as far as I know
Worse for people with other medical problems	Some side effects for some people	Some side effects for some people	Only if you have other problems	Potential side effects	NHS	None
Some seasons can be worse than others. Real flu can be debilitating	It's specially designed for over 65's	None	Feeling protected	Sore arm only	My GP practice would approve	NONE
Very serious illness. As previous answer	Have it at the beginning of the flu season. Specially designed for over 65yrs	No	Peace of mind	No	Family	The undertaker
Can cause very bad health issue	It has an oil in it to help disperse the vaccine throughout the body	Nil	It is more easily absorbed into the body and give very good protection against the flu	Nil	Medical staff, family	No
Having flu is very bad for you	It protects me from flu	No concerns	Protect against flu	No	Family, medical centre staff	No
Can impact heart health if over 65 more than if younger	Specifically designed for over 65's	None	Prevent flu to protect health	None	Family	None that I can think of

What do you know about seasonal flu infection?	What do you know about the adjuvanted seasonal flu vaccine?	What concerns do you have about getting the adjuvanted seasonal flu vaccine?	Some of the good things about getting the adjuvanted seasonal flu	Some of the bad things about getting the adjuvanted seasonal flu	Who would approve of you getting the adjuvanted seasonal flu vaccine?	Who would disapprove of you getting the adjuvanted seasonal flu vaccine?
It confines you to bed and is very unpleasant	Doesn't have many after effects. Seemed fairly innocuous	Possibility of reactions	Don't get flu	You have to appear/wait (possible after effects	Spouse	No. My business!
As before	Specially designed for 65yrs. Designed to increase immunity	No	Peace of mind	None	Family	None
It very bad and makes you very ill	Slightly different for over 65yrs old	No	Have not had flu	No	Family and friends	No
Makes you very unwell. Terrible aches and pains and shivers, bedridden, worse than a cold	Slightly different vaccine that improves absorption	None at all	Hopefully protect me from getting flu	None	Family	None
